# Strategies to improve care for older adults who present to the emergency department: a systematic review

**DOI:** 10.1186/s12913-024-10576-1

**Published:** 2024-02-08

**Authors:** Luke Testa, Lieke Richardson, Colleen Cheek, Theresa Hensel, Elizabeth Austin, Mariam Safi, Natália Ransolin, Ann Carrigan, Janet Long, Karen Hutchinson, Magali Goirand, Mia Bierbaum, Felicity Bleckly, Peter Hibbert, Kate Churruca, Robyn Clay-Williams

**Affiliations:** 1 Australian Institute of Health Innovation, Macquarie University, Level 6, 75 Talavera Road, North Ryde, 2109 Australia; 2 Institute of Medical Sociology, Health Services Research, and Rehabilitation Science (IMVR), University of Cologne, Cologne, Germany; 3 Internal Medicine Research Unit, University Hospital of Southern Denmark, Aabenraa, Denmark; 4 Department of Regional Health Research, University of Southern Denmark, Odense, Denmark; 5 Universidade Federal Do Rio Grande Do Sul, Porto Alegre, RS Brasil; 6 Allied Health and Human Performance, IIMPACT in Health, University of South Australia, Adelaide, 5001 Australia

**Keywords:** Complex system, Urgent healthcare, Quality, Patient safety, Value-based care, Indicators

## Abstract

**Background:**

The aim of this systematic review was to examine the relationship between strategies to improve care delivery for older adults in ED and evaluation measures of patient outcomes, patient experience, staff experience, and system performance.

**Methods:**

A systematic review of English language studies published since inception to December 2022, available from CINAHL, Embase, Medline, and Scopus was conducted. Studies were reviewed by pairs of independent reviewers and included if they met the following criteria: participant mean age of ≥ 65 years; ED setting or directly influenced provision of care in the ED; reported on improvement interventions and strategies; reported patient outcomes, patient experience, staff experience, or system performance. The methodological quality of the studies was assessed by pairs of independent reviewers using The Joanna Briggs Institute critical appraisal tools. Data were synthesised using a hermeneutic approach.

**Results:**

Seventy-six studies were included in the review, incorporating strategies for comprehensive assessment and multi-faceted care (*n* = 32), targeted care such as management of falls risk, functional decline, or pain management (*n* = 27), medication safety (*n* = 5), and trauma care (*n* = 12). We found a misalignment between comprehensive care delivered in ED for older adults and ED performance measures oriented to rapid assessment and referral. Eight (10.4%) studies reported patient experience and five (6.5%) reported staff experience.

**Conclusion:**

It is crucial that future strategies to improve care delivery in ED align the needs of older adults with the purpose of the ED system to ensure sustainable improvement effort and critical functioning of the ED as an interdependent component of the health system. Staff and patient input at the design stage may advance prioritisation of higher-impact interventions aligned with the pace of change and illuminate experience measures. More consistent reporting of interventions would inform important contextual factors and allow for replication.

**Supplementary Information:**

The online version contains supplementary material available at 10.1186/s12913-024-10576-1.

## Introduction

Emergency department (ED) care must adapt to meet current and future demand from an aging and increasingly complex population. Internationally, one in 10 people were aged 65 years or older in 2022; this proportion has been predicted to increase to one in six people by 2050 [1]. The combination of longer life expectancy and limited access to primary healthcare is causing more people to live longer with complex health problems and multiple chronic conditions [2–5]. This, in turn, is driving up the demand for ED care. Older adults attend the ED more frequently than younger people [3]; in Australia, people aged 65 years or older comprise 16% of the population, yet account for 21% of ED presentations [2]. Additionally, 52% of older adults presenting to the ED are admitted to hospital compared to 28% of people overall [2]. Sustaining ED function and high performance to manage this increasing demand for care relies on adaptation across the healthcare system, as well as on strategies within the ED itself.

EDs operate structurally and operationally as part of an integrated health system, purpose-built to provide 24-h access to rapid assessment, stabilisation and referral to hospital inpatient or community-based care [2]. Increasing numbers of ED presentations paired with limited bed capacity can result in longer waiting times and prolonged ED length of stay (LOS). Overcrowding and access block (delay in transferring the person to an admitted hospital ward bed) in the ED have become more common, and are associated with increased medical errors [6, 7], poor patient experiences [8] and poorer outcomes [9, 10] including death [11]. Negative ED outcomes and an inability to influence change may contribute to staff burnout [12, 13]. In response, government policy has endeavoured to better manage unwell older adults in the community to limit their need for hospital care [14]. Notwithstanding these measures, hospital care is required for issues that are beyond the capacity of community providers and so must evolve to meet the needs of patients. Quality improvement strategies that focus on care pathways have predominated over previous decades. In the ED, these include risk stratification screening instruments [15], ortho-geriatric models of care [16] and pathways for condition types such as hip fracture [17]. More recently there has been a movement beyond quality, to deliver value-based healthcare, elevating subjective patient and provider experience together with health system effectiveness [18].

Value-based healthcare considers what matters most for patients, clinicians and the health system [19] with the quadruple aim of providing health services that deliver value across four domains: improved health outcomes, improved patient experiences, improved staff experiences, and better system performance, at a given cost [20]. Moreover, there is an imperative to identify and prioritise high-value interventions that are fit-for-purpose at the local level and interface with, and transform, the interdependent functioning of the overarching health system [18, 21]. Recent syntheses of ED interventions for older adults have been reported [14, 15, 17, 22, 23]. Berning et al. [23] reviewed studies describing interventions that improve patient experience such as consideration of physical needs (e.g. comfort), social needs (e.g. organising transitions to specialist geriatric or primary care services), and minimising waiting times [23]. The authors reported patient ED experience improved with specialist geriatric care and geriatric-friendly care areas that considered their needs (e.g., non-slip floors). Preston et al. [22] undertook an umbrella review of reviews to identify effective ED interventions that have been reported for older people. Most studies reported service metrics, and while there was no individual intervention identified as beneficial, interventions commenced in ED and continued in the community were thought to be the most promising. Notably, most of the reviews had lost details of the primary studies through data abstraction and intervention type and outcomes were variably reported, limiting synthesis [22].

We sought to identify interventions that are effective in targeting aspects of value-based healthcare in the ED for older adults as a foundation for a program to codesign new or adapted models of ED care for this cohort [24]. In this systematic review, we aimed to synthesise the strategies and interventions that have been used to improve care delivery in ED for older adults (aged 65 years and above) that report measures of patient health outcomes, patient experience, staff experience, or system performance.

## Methods

A systematic review was performed and reported in accordance with the Preferred Reporting Items for Systematic Review and Meta-Analyses statement [PRISMA] [25]. The protocol was registered prospectively with Prospero [26].

### Search strategy

A comprehensive search strategy was constructed in consultation with a research librarian. The search terms were broad and included terms to capture articles about the ED, improvements, health and system outcomes, and older adults. Four scholarly databases—CINAHL, Embase, Medline and Scopus – were searched for peer-reviewed articles from inception to December 2022. The search strategy is shown in Supplement 1.

### Eligibility criteria

Peer-reviewed research studies were included in the systematic review if they met the following criteria: (1) participant group had a mean age of 65 years or older; (2) set in the ED or directly influenced provision of care in the ED; (3) reported on improvement interventions; (4) reported measures of patient outcomes, patient experience, staff experience, or system performance. Articles were excluded if they: (1) were not empirical studies (e.g., grey literature, reviews, or perspectives), (2) were undertaken in pre- or post-hospital setting or in a hospital ward other than ED, (3) did not report an intervention, and (4) were published in a language other than English.

### Screening and data extraction

Following the removal of duplicates, each abstract was independently screened by two reviewers according to the prespecified criteria. Included abstracts underwent full-text review by two independent reviewers. Disagreements during both abstract and full-text screening were resolved by discussion or with a third reviewer. Data relating to study characteristics, interventions and outcomes were independently extracted into a specifically designed spreadsheet.

### Quality assessment

The methodological quality of the included peer-reviewed studies was assessed using The Joanna Briggs Institute critical appraisal tools [27]. The tools selected were based on study design and applied independently by pairs of reviewers. Disagreements were resolved through discussion.

### Data synthesis

Data were synthesised using a hermeneutic approach [28] that comprised discussion and interpretation of the various interventions, and iterative sorting of the reviewed studies. Drawing from LT, LR, CC, EA and RCW’s conceptual understanding and domain knowledge, the included studies were categorised as:

1. Comprehensive assessment and multifaceted care: assessment and delivery of the total health care needed or desired by the patient, that is clinically suitable and in line with the patient's health needs
2. Targeted care: interventions specific to the priority presenting health needs of the patient
3. Medication safety: interventions to decrease the frequency of medication errors and/or enhance the safety and quality of medication utilisation
4. Trauma care: interventions initiated following a trauma event to manage the acute needs of the patient.

## Results

### Characteristics of included studies

**Fig. 1:**
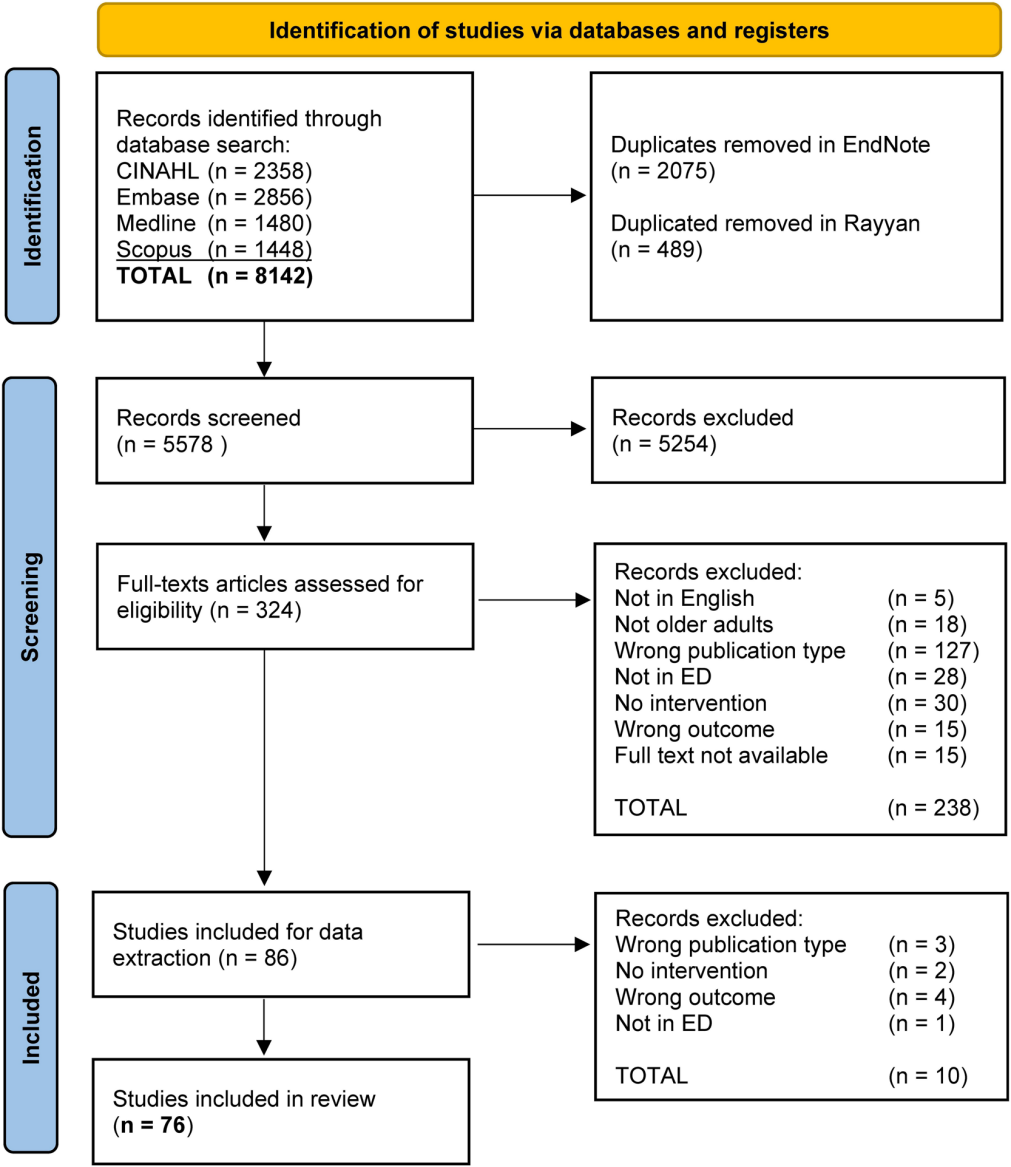
PRISMA flow diagram of studies in the review [29]

Seventy-six studies were included in the review (Fig. 1), comprising 28 pre-post studies, 18 quasi-experimental studies, nine randomised control trials (RCTs), eight cohort studies, five descriptive studies, two cross-sectional studies, two time series studies, two case–control studies, and two qualitative studies. Studies were conducted in the United States of America (*n* = 29), Australia (*n* = 12), Canada (*n* = 10), United Kingdom (*n* = 8), The Netherlands (*n* = 4), Singapore (*n* = 4), France (*n* = 2), Finland (*n* = 1), Germany (*n* = 1), Ireland (*n* = 1), Italy (*n* = 1), Taiwan (*n* = 1), Spain (*n* = 1) and Sweden (*n* = 1). Studies were conducted in one (*n* = 63) or more EDs (*n* = 14).

Risk of bias assessment is reported in Supplement 3.

Thirty-two interventions described comprehensive assessment and multifaceted care for older adults in the ED (Table 1); twenty-one studies aimed to improve system performance by reducing avoidable hospital admissions and/or LOS and/or improve ED flow [30–50]; five aimed to improve patient outcomes [51–55]; five aimed to improve patient experience [56–60]; and one aimed to improve staff experience [61].

**Table 1:** Comprehensive assessment and multi-faceted care interventions for older adults in the ED, by intervention category and level of evidence

Intervention category	Author, Year, Country	Outcome measure	Control	Intervention	*P* value	Level of evidence	Effect
**Comprehensive assessment and multi-faceted care to improve system performance**	Goldberg et al., 2020a, USA [34]	ED LOS (hours), median	5.3	5.0	0.94	II	ne
		Discharged to home, n (%)	39/55 (7.9)	35/55 (63.6)	0.58		ne
		Discharged to skilled nursing facility, n (%)	6/55 (10.9)	10/55 (18.8)	0.58		ne
		Hospital admission, n (%)	10/55 (18.8)	10/55 (18.8)	0.58		ne
	Goldberg et al., 2020b, USA [33]	Fall-related ED visits, IRR (95% CI)	0.34 (0.15, 0.76)		NR	II	+
		All ED visits, IRR (95% CI)	0.47 (0.29, 0.74)		NR		+
		Fall-related hospital admissions, IRR (95% CI)	0.99 (0.31, 3.27)		NR		ne
		All hospital admissions, IRR (95% CI)	0.57 (0.31, 1.04)		NR		ne
	Aldeen et al., 2014, USA [42]	Discharge, %	39.2	55.2	NR	III-2	+
		ED LOS (hours), median (IQR)	5.3 (3.8–7.0)	6.4 (4.9–8.2)	< 0.001*		-
		Inpatient LOS (hours), median (IQR)	90 (48–159)	72 (44–125)	0.07		ne
		3-day ED re-presentation, %	2.7	2.5	NS		ne
		30-day inpatient readmission, %	17.0	13.2	NS		ne
	Arendts et al., 2013, Australia [41]	28-day ED re-attendance, %	14.8	17.9	0.05*	III-2	-
		28-day mortality, %	1.3	1.4	0.85		ne
		One-year unplanned hospitalisations, %	29.5	43.4	< 0.001*		-
		One-year mortality, %	10.2	10.7	0.66		ne
	Beauchet et al., 2021, Canada [39]	ED LOS, β (95% CI)	2.94 (2.00, 3.85)		< 0.001*	III-2	-
		Hospital LOS, β (95% CI)	− 2.07 (− 3.67, − 0.47)		0.01*		+
		Hospital admission, OR (95% CI)	0.92 (0.81, 1.04)		0.182		ne
	Beauchet et al., 2022, Canada [40]	Hospital admission, OR (95% CI)	≤ 0.61 (0.40, 0.93)		0.02*	III-2	+
		ED LOS, β (95% CI)	4.28 (1.13, 7.43)		< 0.01*		-
	Blomaard et al., 2021a, The Netherlands [38]	Compliance with CGA interventions, n (%)	72/343 (21)	114/363 (31.2)	< 0.01*	III-2	+
		ED LOS (min), median (IQR)	202 (133, 290)	196 (133, 265)	0.15		ne
		Hospital admission rate, n (%)	362/920 (40.0)	368/953 (38.9)	0.41		ne
	Bosetti et al., 2020, France [37]	Hospital admission, OR (95% CI)		1.39 (1.05, 1.85)	0.02*	III-2	-
		30-day readmissions, OR (95% CI)		0.65 (0.46, 0.94)	0.02*		+
	Conroy et al., 2014, UK [36]	Hospital admission (≥ 85 years), n (%; 95% CI)	444/638 (69.6; 66.0, 73.1)	461/753 (61.2; 57.7, 64.7)	< 0.001*	III-2	+
		Seven-day readmission (≥ 85 years), n (%); risk ratio (95% CI)	30/638 (4.7); 0.71 (0.42, 1.1)	25/753 (3.3)	NR		ne
		30-day readmission (≥ 85 years), n (%; risk ratio)	79/638(12.4); 0.74(0.55, 1.00)	69/753(9.2)	NR		ne
		90-day readmission (≥ 85 years), n (%; risk ratio)	166/638 (26.0); 0.77 (0.63, 0.93)	150/753(19.9)	NR		+
		LOS (≥ 85 years) (days), mean	8.9	11.1	NR		-
	Ellis et al., 2012, UK [35]	Same day discharge, n (%)	3/212 (1.4)	36/210 (17.1)	< 0.001*	III-2	+
		Direct same day admission to specialty bed, n (%)	149/212 (71)	123/210 (69)	0.02*		-
		Hospital LOS (days), mean (SD)	12.2 (18.63)	12.7 (21.01)	0.78		ne
		7-day readmission, n (%)	14/212 (6.6)	15/210 (7.1)	0.82		ne
		30-day readmission, n (%)	36/212 (17.0)	33/210 (15.7)	0.55		ne
		12-month mortality, n (%)	89/212 (42)	76/210 (36.2)	0.23		ne
		12-month NH admission, n (%)	19/212 (9.0)	24/210 (11.4)	0.69		ne
		12-month living at home, n (%)	104/212 (49.1)	109/210 (51.9)	0.78		ne
	Guttman et al., 2004, Canada [43]	Unscheduled revisits at 8-days post-discharge, risk ratio (95% CI)	0.70 (0.44, 1.10)		NR	III-2	ne
		Unscheduled revisits at 14-days post-discharge, risk ratio (95% CI)	0.80 (0.55, 1.15)		NR		ne
		Satisfaction with the clarity of discharge information, median (interquartile range)	1.5 (1.0, 2.0)	1.7 (1.3, 2.0)	< 0.001*		+
	Heeren et al. 2019 Netherlands [32]	ED LOS (hours), median (range)	19.1 (1.3, 110.3)	12.7 (1.4, 61.2)	< 0.001*	III-2	+
		Hospital admission, n (%)	532/794 (67.0)	620/886 (70.0)	< 0.01*		-
		30-day unplanned ED readmission, n (%)	93/768 (12.1)	112/857 (13.1)	0.28		ne
		Functional decline at 30 days post discharge, n (%)	61/236 (25.9)	52/240 (21.7)	0.04*		+
		Mortality at 90 days post-discharge, n (%)	49/768 (6.4)	48/857 (5.6)	0.73		ne
	Keyes et al., 2014, USA [31]	Hospital admission, relative risk (95% CI)	0.93 (0.89—0.98)			III-2	+
		30-day ED re-attendance, hazard ratio (95% CI)	1.09 (0.95 to 1.23)				ne
		180-day ED re-attendance, hazard ratio (95% CI)	0.99 (0.91 to 1.08)				ne
	Liu et al., 2021, Sweden [30]	Discharged home	566/1,743 (32.5)	306/634 (48.3)	0.04*	III-2	+
		Hospital admission, n (%)	876/1,743 (50.3)	198/634 (31.2)	< 0.01*		+
		Transferred to receiving hospital, n (%)	266/1,743 (15.3)	126/634 (19.9)	0.02*		-
		ED LOS (minutes), median (95% CI)	313 (304, 320)	390 (378, 407)	< 0.01*		-
	Marsden et al., 2022, Australia [47]	*Patients seen in post-GEDI period compared to the pre-GEDI period*				III-2	
		HOSPITAL A					
		Change in hospital LOS (days), mean (95% CI)	1.39 (1.21, 1.59)		NR		-
		Risk of in‑hospital death, prevalence ratio (95% CI)	0.41 (0.24, 0.70)		NR		+
		Same cause ED representation, hazard ratio (95% CI)	NR		NR		
		Any cause representation, hazard ratio (95% CI)	0.89 (0.84, 0.95)		NR		+
		Readmission any reason, hazard ratio (95% CI)	1.07 (0.90, 1.28)		NR		ne
		HOSPITAL B					
		Change in hospital LOS (days), mean (95% CI)	‑0.39 (‑0.54, ‑0.23)		NR		+
		Risk of in‑hospital death, prevalence ratio (95% CI)	0.66 (0.37, 1.16)		NR		ne
		Same cause ED representation, hazard ratio (95% CI)	0.96 (0.87,1.07)		NR		ne
		Any cause representation, hazard ratio (95% CI)	0.98 (0.92,1.05)		NR		ne
		Readmission any reason, hazard ratio (95% CI)	1.10 (0.88, 1.39)		NR		ne
		*Patients seen by GEDI compared to those not seen by GEDI in the post-GEDI period*					
		HOSPITAL A					
		Change in hospital LOS (days), mean (95% CI))	0.63 (0.41, 0.87)		NR		-
		Risk of in‑hospital death, prevalence ratio (95% CI)	0.43 (0.15, 0.98)		NR		+
		Same cause ED representation, hazard ratio (95% CI)	NR		NR		
		Any cause representation, hazard ratio (95% CI)	1.00 (0.92, 1.09)		NR		ne
		Readmission any reason, hazard ratio (95% CI)	1.21 (0.96, 1.53)		NR		ne
		HOSPITAL B					
		Change in hospital LOS (days), mean (95% CI)	‑0.12 (‑0.29, 0.05)		NR		+
		Risk of in‑hospital death, prevalence ratio (95% CI)	0.84 (0.41, 1.59)		NR		ne
		Same cause ED representation, hazard ratio (95% CI)	1.45 (1.29, 1.64)		NR		-
		Any cause representation, hazard ratio (95% CI)	1.60 (1.49, 1.73)		NR		-
		Readmission any reason, hazard ratio (95% CI)	1.47 (1.14, 1.89)		NR		-
	Southerland et al., 2018, UK [48]	Hospital admission	25.7%	25.8%	NS	III-2	ne
		LOS in observation (hours), mean (range)	14.3 (1.7, 42.7)	15.3 (1.1, 35.5)	NS		ne
	Wallis et al., 2018, Australia [50]	Likelihood of discharge, (Hazard ratio, 95% CI)	1.2 (1.1, 1.2)		NR	III-2	+
		Reduced ED LOS, (Hazard ratio, 95% CI)	1.3 (1.2, 1.4)		NR		+
		Reduced hospital LOS, (Hazard ratio, 95% CI)	1.0 (0.9, 1.1)		NR		ne
		Risk of death, (Hazard ratio, 95% CI)	1.0 (0.2, 4.4)		NR		ne
		28-day ED representation, (Hazard ratio, 95% CI)	1.2 (0.9, 1.5)		NR		ne
		Cost savings per ED presentation (AU$), mean (95% CI)	35 (21, 49)		NR		+
		Cost savings per hospital admission (AU$), mean (95% CI)	1,469 (1,105, 1,834)		NR		+
	Warburton et al., 2005, Canada [49]	*Not screened vs screened as high-risk, received complete referrals*				III-2	
		LOS (days), median	8	6	NR		ne
		30-day revisit ED, %	18	16	NR		ne
		Hospital admission, %	9	13	NR		ne
		30-day revisit ED and/or admission, %	24	21	NR		ne
		30-day multiple encounters, %	9	8	NR		ne
		*Not screened vs screened as high-risk, received partial or no referrals*					
		LOS (days), median	8	8	NR		ne
		30-day revisit ED, %	18	21	NR		ne
		Hospital admission, %	9	21	NR		-
		30-day revisit ED and/or admission, %	24	35	NR		ne
		30-day multiple encounters, %	9	9	NR		ne
		*Not screened vs screened low-risk*					
		LOS (days), median	8	4	NR		ne
		30-day revisit ED, %	18	7	NR		ne
		Hospital admission, %	9	2	NR		ne
		30-day revisit ED and/or admission, %	24	9	NR		ne
	Dresden et al., 2020, USA [44]	*Readmission during ED visit (average incremental effect)*				III-3	
		Hospital A, % difference (95% CI)		-10.1 (-20.9, 0.8)	NR		ne
		Hospital B, % difference (95% CI)		-17.4 (-25.2, -9.6)	< 0.05*		+
		Hospital C, % difference (95% CI)		-2.5 (-13.7, 8.8)	NR		ne
		*ED discharge, but subsequent readmission within 30 days of prior hospitalisation (average incremental effect)*					
		Hospital A, % difference (95% CI)		4.4 (-1.4, 10.3)	NR		ne
		Hospital B, % difference (95% CI)		1.2 (-2.3, 4.6)	NR		ne
		Hospital C, % difference (95% CI)		3.1 (-3.0, 9.2)	NR		ne
		*30-day inpatient readmission (average incremental effect)*					
		Hospital A, % difference (95% CI)		-5.6 (-16.3, 5.1)	NR		ne
		Hospital B, % difference (95% CI)		-16.2(-24.0, -8.5)	< 0.05*		+
		Hospital C, % difference (95% CI)		0.63 (-10, 11.3)	NR		ne
	Keene et al., 2022, America [45]	Discharge, % (OR; 95% CI)	29 (2.06; 1.73,2.47)	54	NR	III-3	+
		ED LOS (hours), mean	4.62	4.94	< 0.01*		-
		Hospital LOS (days), mean	5.54	4.50	< 0.01*		+
	Silvester et al., 2014, UK [46]	Reduction in bed occupancy, mean (95% CI)		-20.4 (-39.6, − 1.2)	0.04*	III-3	+
		In-hospital mortality, % (95% CI); OR (95% CI)	11.4 (10.4, 12.4); 0.8 (0.6, 1.0)	9.15 (7.6, 10.7)	0.06		ne
		28-day readmission rate, % (OR; 95% CI)	17.1 (0.9; 0.8, 1.2)	16.3	0.61		ne
**Comprehensive assessment and multi-faceted care to improve patient outcomes**	Vivanti et al., 2015, Australia [51]	Weight change (kg), mean (SD)	− 1.1 (4.6)	0.8 (3.7)	NS	II	ne
		LOS (days), median (range)	6 (2, 59)	4.5 (1, 60)	NS		ne
		EDQ5 quality of life, mean (SD)	0.1 (16.4)	14.4 (29)	NS		ne
		Depression (EDQ5), mean (SD)	1.4 (6.9)	0.9 (3.0)	NS		ne
		Further decline in nutritional status, n (%)	0/9 (0)	1/10 (10)	NS		ne
	Huded et al., 2022, USA [53]	Consults to pharmacy, n (%)	195/725 (26.9)	315/725 (43.4)	< 0.001*	III-2	+
		Consults to social work, n (%)	132/725 (18.2)	399/725 (55.0)	< 0.001*		+
		Referrals to Geriatrics, n (%)	18/725 (5.8)	64/725 (17.7)	< 0.001*		+
		Referrals to Home Based Primary Care, n (%)	24/725 (7.8)	110/725 (30.4)	< 0.001*		+
		Hospital admission, n (%)	417/725 (57.5)	363/725 (50.1)	< 0.01*		+
		30-day hospital admissions, n (%)	464/725 (64.0)	412/725 (56.8)	< 0.001*		+
		ED LOS (hours), n (%)	5.4/725 (2.6)	5.4/725 (2.2)	0.85		ne
		72-h ED representations, n (%)	16/725 (2.2)	23/725 (3.2)	0.25		ne
	Hullick et al., 2018, Australia [52]	Screening of cognition, n (%)	1/63 (1.5)	24/63 (38)	< 0.001*	III-2	+
		Review of pain, n (%)	18/63 (29)	47/63 (75)	< 0.001*		+
		Given food or fluids, n (%)	8/63 (13)	31/63 (49)	< 0.001*		+
		Orientation, n (%)	0/63 (0)	32/63 (51)	< 0.001*		+
		Toileting, n (%)	0/63 (0)	21/63 (33)	< 0.001*		+
		Mobilisation, n (%)	0/63 (0)	26/63 (41)	< 0.001*		+
		Pressure care, n (%)	3/63 (4.8)	19/63 (30)	< 0.001*		+
		ED LOS (minutes), mean (SD)	412 (257)	524 (278)	NR		-
		Discharged from ED, n (%)	5,660/8,287 (68)	1,161/4563 (25)	NR		-
		Admitted to hospital, n (%)	2,627/8,287 (32)	3,402/4563 (75)	NR		-
		Clinicians’ experiences of the OPTA role		Mixed responses and support for the OPTA role			±
		30-day multiple encounters, %	9	2	NR		ne
	Lee et al., 2001, Canada [55]	*Discharged home vs admitted to hospital*				III-3	
		Lives with others, %	22.5	7.5	0.4		
		SMAF disability score, mean	16.6	25.4	< 0.01*		
		SMAF handicap score, mean	6.3	12.6	< 0.001*		
	Ngian et al., 2008, Australia [54]	*Documentation of pre-morbid:*				IV	
		Functional impairment, n (%)	49/103 (48)	68/103 (66)	< 0.01*		+
		Cognitive impairment, n (%)	30/103 (29)	73/103 (71)	< 0.01*		+
		Mobility impairment, n (%)	46/103 (45)	85/103 (83)	< 0.01*		+
		*Documentation and assessment at presentation for:*					
		Functional impairment, n (%)	1/103 (1)	36/103 (36)	< 0.01*		+
		Cognitive impairment, n (%)	22/103 (21)	70/103 (68)	< 0.01*		+
		Mobility impairment, n (%)	9/103 (9)	52/103 (51)	< 0.01*		+
Comprehensive assessment and multi-faceted care to improve patient experience	Corbett et al., 2005, Australia [56]	Hospital admission, n (%)	8,170/40,510 (20.2)	8,699/48,238 (18.0)	< 0.01*	III-2	+
		AQoL social relationships score, mean (SD)	0.6 (0.3)	0.9 (0.1)	< 0.01*		+
		AQoL Psychological wellbeing score, mean (SD)	0.6 (0.3)	0.9 (0.1)	< 0.01*		+
		AQoL utility score, mean (SD)	0.3 (0.2)	0.6 (0.2)	< 0.01*		+
	Argento et al., 2014, USA [57]	ED LOS (minutes), mean	401	360	NR	III-3	+
		Discharge time (minutes), mean	302	258	NR		+
		Patient satisfaction, mean	69.9	93.8	NR		+
	McGrath et al., 2019, UK [59]	> *75 years presenting to ED by ambulance*				III-3	
		Clinical Frailty Scale completed, %		73	NR		+
		> *75 years attending ED through any route*					
		Clinical Frailty Scale completed, %		47	NR		ne
		Hospital admission	50.7	49.2	NR		ne
		*Staff satisfaction survey*					
		Felt confident about using the Rockwood CFS, n (%)		17/22 (77)	NR		+
		Felt the frailty team was beneficial to patient flow through ED, n (%)		22/22 (100)	NR		+
		*Patient satisfaction*					
		Happy with the experience of the frailty pathway, n (%)		9/10 (90)	NR		+
		Feeling the additional time spent to complete a CGA was acceptable, n (%)		8/10 (80)	NR		+
		Experience as better or the same as any previous ED attendance, n (%)		10/10 (100)	NR		+
	Palonen et al., 2015, Finland [58]	*Pre-discharge confidence*				IV	
		Did not need more information, OR (95% CI)		2.7 (1.0, 7.5)	0.05		ne
		*No worries after discharge*					
		Did not need more information, OR (95% CI)		4.8 (1.9, 11.8)	0.001*		+
		*No unexpected problems after discharge*					
		Did not need more information, OR (95% CI)		3.8 (1.5, 9.6)	< 0.01*		+
		*Overall readiness two weeks after discharge*					
		Did not need more information, OR (95% CI)		10.4 (3.7, 29.2)	< 0.001*		+
		Received discharge education		3.7 (1.3, 10.3)	0.01*		+
	Blomaard et al., 2021b, The Netherlands [60]	Recall of screening administration		Noticed little of the screening administration during triage and screening was considered as a normal part of ED care		N/A	+
		Experienced consequences of screening		None of the participants had a negative attitude towards screening or thought that screening is age discrimination			+
		Added value of screening		Most participants believed that geriatric screening contributes to assessing older adults holistically, recognising geriatric problems early and comforting patients with communication and attention			+
Comprehensive assessment and multi-faceted care to improve staff experience	O'Grady et al., 1996, Australia [61]	ED LOS		Not impacted		N/A	ne
		Likely admissions avoided, %		33			+
		Waiting time		Not impacted			ne
		LOS		Not impacted			ne
		Patient satisfaction, %		85			+
		GPs satisfied with the Quick Response Program concept, %		79			+
		GPs satisfied with arrangements made for their Quick Response Program patients, %		71			+

Characteristics of interventions and study populations reported in Supplement 1. *ADL* Activities of daily living, *AQoL* Assessment of Quality of Life, *CCI* Charlson comorbidities index, *CFS* Clinical Frailty Scale, *CI* confidence interval, *EAU* emergency assessment unit, *ED* Emergency Department, *FI* frailty index, *GEDI* Geriatric Emergency Department Intervention, *GP* General Practitioner, *IQR* interquartile range, *IRR* incidence rate ratio, *LOS* length of stay. ne: no effect, *MBI* Modified Barthel Index, *MMSE* Mini-Mental State Examination, *NR* not reported, *NS* not significant, *N/A* not applicable, *OPTA* Older Person Technical Assistant, *OR* odds ratio, *PT* physical therapy, *SD* standard deviation, *SMAF* Functional Autonomy Measurement System. *TCN* transitional care nurse, + positive effect,—negative effect, *β* coefficient beta

*denotes statistical significance

Twenty-seven studies described targeted care for older adults in the ED (Table 2): fourteen studies aimed to improve system performance [62–75]; seven studies aimed to improve patient outcomes [76–82]; three studies aimed to improve patient experience [83–85]; and three aimed to improve staff experience [86–88].

**Table 2:** Targeted care interventions for older adults in the ED, by intervention category and level of evidence

Intervention category	Author, Year, Country	Outcome measure	Control	Intervention	P value	Level of evidence	Effect
**Targeted care to improve system performance**	Basic et al., 2005, Australia [64]	Admission to the hospital, OR (95% CI)	0.7 (0.3, 1.7)		NR	II	ne
		Hospital LOS, hazard ratio (95% CI)	1.1 (0.7, 1.5)		NR		ne
		Functional decline during the hospitalisation, OR (95% CI)	1.3 (0.5, 3.3)		NR		ne
	McCusker et al., 2003a, Canada [62]	4-month decline in functional status or death, OR (95% CI)		0.5 (0.3—0.9)	NR	II	ne
		Depressive symptom change, OR (95% CI)		-0.5 (-1.3, 0.3)	NR		ne
		4-month difference in health care costs after index visit, CA$ (95% CI)		-387 (-1411, 638)	NR		ne
	McCusker et al., 2003b, Canada [63]	Referrals to primary physician, OR (95% CI)	1.9 (1.0, 3.4)			II	+
		Compliance with referrals, OR (95% CI)	1.2 (0.7, 2.3)				ne
		30-day ED re-presentations, OR (95% CI)	1.6 (1.0, 2.6)				-
	Ageron et al., 2016, France [70]	Fall-related ED attendance, n (%); relative risk ratio (95% CI)	46/144 (32); 1.3 (0.9, 1.7)	52/130 (40)	NR	III-2	ne
		Fall-related hospital admission, n (%); relative risk ratio (95% CI)	19/144 (13); 1.1 (0.6, 2.0)	19/130 (15)	NR		ne
		1-year mortality	29/144 (20); 1.0 (0.7, 1.6)	27/130 (21)	NR		ne
		Mortality during hospital stay	50/2,426 (2.1)	61/2,684 (2.3)	0.61		ne
		Fall recurrence within 1 month in older adults discharged, not living in an aged care facility, and without cognitive impairment	29/2,426 (3.6)	17/2,684 (2.0)	0.05		ne
		Hospital LOS (days), mean (SD)	13.1 (12.7)	11.6 (9.1)	< 0.01*		+
	Brymer et al., 2001, UK [73]	*Change in assessment practices of ED nurses*				III-2	
		Routinely assesses for depression		↑	< 0.001*		+
		Routinely assesses for altered mental status		↑	< 0.01*		+
		Routinely assesses for dementia		no change	0.54		ne
		Routinely asks what client weighs		no change	0.10		ne
		Routinely asks if unplanned weight loss		no change	0.23		ne
		Routinely asks if there is assistance in the home			0.05		ne
	Chong et al. 2021, Singapore [66]	1-month rehospitalisation, IRR (95% CI)	1.5 (0.5, 4.4)		0.42	III-2	ne
		3-month rehospitalisation, IRR (95% CI)	0.9 (0.5, 1.7)		0.74		ne
		6-month rehospitalisation, IRR (95% CI)	0.8 (0.5, 1.3)		0.33		ne
		1-month ED re-attendance, IRR (95% CI)	1.7 (0.6, 4.7)		0.29		ne
		3-month ED re-attendance, IRR (95% CI)	0.9 (0.4, 1.8)		0.74		ne
		6-month ED re-attendance, IRR (95% CI)	0.6 (0.3, 1.0)		0.08		ne
		Mortality (over study period), IRR (95% CI)	0.3 (0.1, 1.3)		0.11		ne
		Institutionalisation (over study period), IRR (95% CI)	0.8 (0.2, 3.9)		0.82		ne
		3-month fall, IRR (95% CI)	0.4 (0.1, 1.9)		0.23		ne
		6-month fall, IRR (95% CI)	0.4 (0.1, 1.5)		0.18		ne
		1-month polypharmacy (≥ 5 medications), IRR (95% CI)	1.95 (0.7, 5.7)		0.22		ne
		3-month polypharmacy (≥ 5 medications), IRR (95% CI)	0.9 (0.3, 2.5)		0.83		ne
		6-month polypharmacy (≥ 5 medications), IRR (95% CI)	0.9 (0.4, 2.3)		0.89		ne
		1-month increase in CFS score from baseline (≥ 5 medications), IRR (95% CI)	0.4 (0.2, 1.0)		0.06		ne
		3-month increase in CFS score from baseline (≥ 5 medications), IRR (95% CI)	0.4 (0.2, 1.0)		0.05		ne
		6-month increase in CFS score from baseline (≥ 5 medications), IRR (95% CI)	0.3 (0.1, 0.9)		0.04*		+
		1-month progression in CFS category from baseline (≥ 5 medications), IRR (95% CI)	0.2 (0.1, 0.5)		< 0.001*		+
		3-month progression in CFS category from baseline (≥ 5 medications), IRR (95% CI)	0.1 (0.0, 0.4)		< 0.001		+
		6-month progression in CFS category from baseline (≥ 5 medications), IRR (95% CI)	0.2 (0.1, 0.7)		0.01*		+
	Miller et al., 1996, USA [67]	ED LOS (minutes), mean	292	231	< 0.001*	III-2	+
		Subsequent visits to emergency departments	unclear	unclear	0.06		ne
		Number of new dental or social services initiated per patient, mean	1.5	1.7	NR		ne
		Advance care directives, %	2.9	6.7	0.07		ne
		3-month mortality, %	9.7	9.3	NR		ne
	O' Keeffe et al., 2020, Ireland [71]	Hospital admission, n (%)	8 (14)	7 (9)	0.11	III-2	ne
	Liberman et al., 2018, USA [72]	Identified as having advanced illness in the ED, %	0.0	90.2	< 0.001*	III-2	+
		Received an ED-led Goals of Care discussion (%)	0.0	83.6	< 0.001*		+
		Patients referred to hospice from the ED (%)	0.0	39.3	< 0.001*		+
	Newton-Brown et al., 2014, Australia [74]	Received nerve block, n (%; 95% CI)	17/70 (24.3; 15.8, 35.5)	35/66 (53.0; 41.2, 64.6)	< 0.01*	III-2	+
		Nerve block documented in medical record, n (%; 95% CI)	12/17 (70.6; 46.9, 86.7)	33/35 (94.3; 81.4, 98.4)	< 0.01*		+
	Scarpazza et al., 2008, Italy [75]	Successfully treated, n (%)		54/62 (87.1)		III-3	+
		Hospital LOS, mean (SD)		13.7 (5.1)			
	Basic et al. 2002a, Australia [65]	Not admitted to hospital		142/469 (30.2)		N/A	
	Puig Campmany et al. 2019, Spain [68]	Hospital admissions, %	12%	11.3%	NR	N/A	+
	Tousignant-Laflamme et al. 2015, Canada [69]	Implementation facilitators		Easy access to the list of patients admitted in the ED; sufficient time to provide PT with help of 4th year physiotherapy student; good collaboration with nursing staff		N/A	
		Implementation barriers		Fast transfer of patients from ED and lack of space to provide optimal PT; lack of time to complete screening document; lack of communication between shifts, nursing staff turnover			
		Assessed by physiotherapist in the ED after screening, n (%)		20/187			
		Assessed by physiotherapist and received PT treatment in the ED and developed immobilization syndrome, n (%)		0/9			
		Assessed by physiotherapist and did not receive PT treatment in the ED and developed immobilization syndrome		2/11			
**Targeted care to improve patient outcomes**	Shaw et al., 2003, UK [81]	Falls one-year post intervention, n (%); relative risk ratio (95% CI)	115/144 (80); 0.9 (0.8, 1.1)	96/130 (74)	NR	II	ne
	Chong et al. 2022, Singapore [78]	3-month MBI, mean (SD)	90.2 (17.4)	95.5 (7.8)	0.05	III-2	ne
		6-month MBI, mean (SD)	88.5 (19.5)	94.5 (11.2)	0.04*		+
		12-month MBI, mean (SD)	90.2 (18.0)	93.6 (15.2)	0.3		ne
		3-month instrumental ADLs, mean (SD)	5.1 (2.4)	5.7 (1.9)	0.15		ne
		6-month instrumental ADLs, mean (SD)	4.9 (2.6)	5.4 (2.4)	0.22		ne
		12-month instrumental ADLs, mean (SD)	4.7 (2.5)	5.7 (2.4)	0.05		ne
		3-month CFS, mean (SD)	4.9 (1.0)	4.7 (0.8)	0.26		ne
		6-month CFS, mean (SD)	4.8 (0.9)	4.6 (0.7)	0.19		ne
		12-month CFS, mean (SD)	5.0 (1.0)	4.8 (0.9)	0.18		ne
		3-month FI, mean (SD)	0.23 (0.08)	0.22 (0.06)	0.32		ne
		6-month FI, mean (SD)	0.25 (0.07)	0.22 (0.06)	0.02*		+
		12-month FI, mean (SD)	0.25 (0.09)	0.23 (0.07)	0.02*		+
		3-month SARC-F, mean (SD)	3.8 (2.4)	3.3 (2.2)	0.23		ne
		6-month SARC-F, mean (SD)	4.1 (2.8)	3.1 (2.4)	0.04*		+
		12-month SARC-F, mean (SD)	3.7 (2.8)	3.0 (2.4)	0.22		ne
		3-month CCI, mean (SD)	1.9 (2.0)	2.3 (2.3)	0.42		ne
		6-month CCI, mean (SD)	1.9 (1.9)	2.4 (2.2)	0.28		ne
		12-month CCI, mean (SD)	2.0 (2.0)	1.8 (1.7)	0.62		ne
	Foo et al., 2012, Singapore [76]	3-month falls, n (%); IRR (95% CI)	20/93 (11.6); 0.91 (0.44, 1.90)	23/177 (7.3)	NR	III-2	ne
		3-month mortality, n (%); IRR (95% CI)	1/88 (0.58); 0.81 (0.06, 11.81)	2/162 (0.63)	NR		ne
		3-month ED re-attendance, n (%); IRR (95% CI)	49/169 (28.5); 0.58 (0.42, 0.81)	54/293 (17.1)	NR		+
		3-month hospitalisation, n (%); IRR (95% CI)	40/169 (23.3); 0.61 (0.41, 0.92)	48/293 (15.2)	NR		+
	Foo et al. 2014, Singapore [77]	MBI at baseline, n (difference)	500/500 (0)	280/280 (0)	0.1	III-2	
		ADL at baseline, n (difference)	500/500 (0)	280/280 (0)	< 0.01*		
		MBI difference at 3 months, n (difference)	479/500 (-0.25)	269/280 (0)	< 0.01*		+
		ADL difference at 3 months, n (difference)	479/500 (-0.33)	269/280 (0.53)	< 0.01*		+
		MBI difference at 6 months, n (difference)	469/500 (-0.53)	260/280 (0.03)	< 0.01*		+
		ADL difference at 6 months, n (difference)	469/500 (-1.24)	260/280 (0.6)	< 0.01*		+
		MBI difference at 9 months, n (difference)	439/500 (-0.78)	248/280 (-0.08)	< 0.01*		+
		ADL difference at 9 months, n (difference)	439/500 (-2.02)	248/280 (0.63)	< 0.01*		+
		MBI difference at 12 months, n (difference)	423/500 (-0.99)	234/280 (-0.24)	< 0.01*		+
		ADL difference at 12 months, n (difference)	423/500 (-2.57)	234/280 (0.45)	< 0.01*		+
		*Intention-to-treat analysis*					
		3-month ED attendance, OR (95%CI)	0.9 (0.6, 1.2)		NR		ne
		6-month ED attendance, OR (95%CI)	0.8 (0.6, 1.1)		NR		ne
		9-month ED attendance, OR (95%CI)	0.7 (0.6,1.0)		NR		ne
		12-month ED attendance, OR (95%CI)	0.8 (0.6,1.0)		NR		ne
		3-month hospitalisation, OR (95%CI)	0.9 (0.6, 1.2)		NR		ne
		6-month hospitalisation, OR (95%CI)	0.8 (0.6, 1.1)		NR		ne
		9-month hospitalisation, OR (95%CI)	0.8 (0.6, 1.0)		NR		ne
		12-month hospitalisation, OR (95%CI)	0.8 (0.6, 1.0)		NR		ne
		*Per protocol analysis*					
		3-month ED attendance, OR (95%CI)	0.8 (0.6, 1.1)		NR		ne
		6-month ED attendance, OR (95%CI)	0.7 (0.5, 0.9)		NR		+
		9-month ED attendance, OR (95%CI)	0.6 (0.5, 0.9)		NR		+
		12-month ED attendance, OR (95%CI)	0.7 (0.4, 0.8)		NR		+
		3-month hospitalisation, OR (95%CI)	0.8 (0.6, 1.2)		NR		ne
		6-month hospitalisation, OR (95%CI)	0.8 (0.6, 1.1)		NR		ne
		9-month hospitalisation, OR (95%CI)	0.7 (0.5, 0.9)		NR		+
		12-month hospitalisation, OR (95%CI)	0.8 (0.6, 1.0)		NR		ne
	Hogan et al., 2016, USA [80]	Change in pain score (initial to follow-up), median (IQR)	-1.0 (-3.0, 0.0)	-3.0 (-5.0, -1.0)	< 0.001*	III-2	+
		Change in pain score (initial to final), median (IQR)	0.0 (-2.0, 0.0)	-5.0 (-7.0, -2.0)	< 0.001*		+
		Received pain score in triage, n (%)	427 (85.6)	294 (85.7)	0.95		ne
		Received medicine after initial pain score, n (%)	320 (64.1)	291 (84.8)	< 0.001*		+
		Time to first medication after arrival (minutes), median (IQR)	118 (64, 240)	118 (61, 213)	0.70		ne
		Reassessment after first medication, n (%)	166/499 (51.9)	240/343 (82.5)	< 0.01*		+
		Time to first reassessment after first medication (minutes), median (IQR)	86.0 (20.0, 199.0)	65.0 (27.0, 175.0)	0.60		ne
	Lesser et al., 2018, USA [79]	30-day ED re presentations, (OR)	0.7		< 0.001*	III-2	+
		60-day ED re presentations, (OR)	0.7		< 0.001*		+
	Mahony et al., 2008, USA [82]	ED attendance 12-months before index visit, %		59		N/A	
		ED attendance 12-months after index visit, %		45			ne
		Satisfaction with symptom control post ED-discharge, n (%)		14/20 (69)			+
**Targeted care to improve patient experience**	McCusker et al., 2001, Canada [83]	*Change at 4 months compared to baseline*				II	
		Functional decline, OR (95% CI)	0.5 (0.3, 0.9)				+
		Depressive symptoms, OR (95% CI)	-0.5 (-1.3, 0.3)				ne
		Caregiver mental health, OR (95% CI)	-2.2 (-5.9, 1.6)				ne
		Caregiver satisfaction, OR (95% CI)	0.71 (-0.6, 2.0)				ne
		Patient satisfaction, OR (95% CI)	0.66 (-0.24, 1.55)				ne
	Boucher et al. 2019, Canada [84]	Adjusted Treatment Acceptability and Preferences scale scores (Research Assistant evaluation vs patient self-assessment), mean	2.20	2.36	0.08	III-1	+
	Liberman et al., 2020, USA [85]	30-day ED revisit, mean (%)	0.22	0.20	0.34	III-2	ne
		Hospital admission at 30-day revisit, n (%)	35 (57)	23 (40)	0.01		+
		*Patient satisfaction*					
		Found the Geriatric and Palliative-ED Specialist helpful in providing support and resources, %		220/242 (91)			+
		Think EDs should have a Geriatric and Palliative-ED team to consult patients and caregivers		219/242 (90)			+
**Targeted care to improve staff experience**	Arendts et al., 2020, Australia [87]	ED discharge, %	46	66	0.001*	III-2	+
		ED LOS (hours), mean	6.5	3.6	< 0.001*		+
		Hospital LOS (days), mean	6	2	< 0.001*		+
		28-day re-presentation rate to the ED	NR	NR	NR		ne
		*Staff views on pathway*					
		Staff aware of pathway, n (%)		34/34 (100)	NR		+
		Believed pathway improved overall care and improved knowledge of falls patients,		19/34 (56)	NR		+
	Desy et al., 2008, USA [88]	Total knowledge score, mean (SD)	23.9 (2.5)	27.2 (1.4)	< 0.001*	III-2	+
		Self-rated ability to provide geriatric care			NS		ne
		*Use of geriatric assessment tools*					
		MMSE, %		↑	0.01*		+
		Pain assessment, %		↓	0.03*		-
		Braden scale for predicting pressure sore risk, %		↓	0.01*		-
		Urinary incontinence assessment, %		↑	< 0.01*		+
		Falls risk assessment, %		↑	0.01*		+
		Pain assessment in patients with dementia, %		↑	0.01*		+
		*Incorporated knowledge learned 3-months after attending course*					
		Completely, %		37			+
		Somewhat, %		51			+
		EDs incorporating geriatric protocols of care, %	12	21	< 0.01*		+
	Elliott et al. 2017, UK [86]	Ideal tool characteristics		Tools should be multidimensional, short (< 5 min), and validated		N/A	
		Timing between CFS, ISAR, PRISMA-7, and Silver Code		No significant differences between professions for the time taken to complete an assessment			+
		Ease of use between CFS, ISAR, PRISMA-7, and Silver Code		No significant differences in ease of use			+
		Agreement with clinical judgement between CFS, ISAR, PRISMA-7, and Silver Code		Good agreement between participants’ clinical judgement			+

Characteristics of interventions and study populations reported in Supplement 1. *ADL* Activities of daily living, *AQoL* Assessment of Quality of Life, *CCI* Charlson comorbidities index, *CFS* Clinical Frailty Scale, *CI* confidence interval, *EAU* emergency assessment unit, *ED* Emergency Department, *FI* frailty index, *GEDI* Geriatric Emergency Department Intervention, *GP* General Practitioner, *IQR* interquartile range, *IRR* incidence rate ratio, *LOS* length of stay, *ne* no effect, *MBI* Modified Barthel Index, *MMSE* Mini-Mental State Examination, *NR* not reported, *NS* not significant, *N/A* not applicable, *OPTA* Older Person Technical Assistant, *OR* odds ratio, *PT* physical therapy, *SD* standard deviation, *SMAF* Functional Autonomy Measurement System, *TCN* transitional care nurse. + positive effect,—negative effect, *β* coefficient beta

*denotes statistical significance

Five studies described interventions for medication safety (Table 3): four studies aimed to improve system performance [89–92]; one study aimed to improve staff performance [93]. Twelve studies described intervention to deliver better trauma care (Table 4): all twelve studies aimed to improve system performance [94–105]. The characteristics of the included studies are detailed in Supplement 2, including the Study aims and intervention description.

**Table 3:** Medication management interventions for older adults in ED, by intervention category and level of evidence

Intervention category	Author, Year, Country	Outcome measure	Control	Intervention	P value	Level of evidence	Effect
Medication management to improve system performance	Liu et al., 2019, Taiwan [91]	Reduction in major polypharmacy (≥ 10 medications) at hospital discharge compared with on admission to the ED, %	-65.3	-79.4	< 0.001*	III-2	+
		Reduction in PIMs at hospital discharge compared with on admission to the ED, %	-49.1	-67.5	< 0.001*		+
		Number of medications, mean (SD)	12.5 (2.7)	6.9 (3)	< 0.001*		+
	Matz et al., 2021, Germany [92]	Immediate drug interventions/recommendations for pre-existing medications, mean (SD)	1.24 (1.71)	3.28 (2.22)	< 0.001*	III-2	+
		Medications discontinued, mean (SD)	0.60 (1.25)	1.74 (1.32)	< 0.001*		+
		Medications commenced, mean (SD)	0.50 (0.93)	0.86 (0.93)	0.004*		+
		Altered dose, mean (SD)	0.14 (0.35)	0.88 (0.82)	0.001*		+
		FORTA drugs, n (%)	8/65 (12.3)	35/65 (53.9)	< 0.001*		+
	Stevens et al., 2017, USA [89]	PIMs prescribed—Site 1, % (SD)	11.9 (1.8)	5.1 (1.4)	< 0.001*	III-2	+
		PIMs prescribed—Site 2, % (SD)	8.2 (0.8)	4.5 (1.0)	< 0.001*		+
		PIMs prescribed—Site 3, % (SD)	8.9 (1.9)	6.1 (1.7)	< 0.001*		+
		PIMs prescribed—Site 4, % (SD)	7.4 (1.7)	5.7 (0.8)	0.04*		+
	Vaughan et al., 2021, USA [90]	PIMs prescribed—Site 1, % (95% CI)	5.6 (5.0, 6.3)	5.1 (4.7, 5.5)	0.02*	III-2	+
		PIMs prescribed—Site 2, % (95% CI)	5.8 (5.0, 6.6)	5.4 (4.8, 6.0)	0.62		ne
		PIMs prescribed—Site 3, % (95% CI)	7.3 (6.4, 9.2)	7.5 (6.6, 8.4)	0.64		ne
Medication management to improve staff experience	Moss et al., 2019, USA [93]	Self-reported confidence in prescribing for older adults, %	80	100	0.005*	III-2	+
		PIMs prescribed to older adults by physician residents, rate ratio (95% CI)	0.73 (0.63, 0.85		< 0.001*		+

Characteristics of interventions and study populations reported in Supplement 1. *CI* confidence interval, *EQUiPPED* Enhancing Quality of Provider Practices for Older Adults in the Emergency Department, *FORTA* Fit for the aged, *ne* no effect, *PIM* potentially inappropriate medications, *SD* standard deviation, + positive effect

*denotes statistical significance

**Table 4:** Trauma care interventions for older adults in ED, by level of evidence

Intervention category	Author, Year, Country	Outcome measure	Control	Intervention	P value	Level of evidence	Effect
Trauma care interventions to improve system performance	Callahan et al., 2020, USA [104]	Received trauma activation, %	19.9	74.9	< 0.001*	III-2	+
		Percentage discharged directly home without injury	4.3	44	< 0.001*		+
		Critical ED disposition and failed to receive trauma activation, %	65.1	23.5	< 0.001*		+
		Traumatic intracranial haemorrhage and failed to receive a trauma activation, %	70.7	27.3	< 0.001*		+
		Hospital LOS (days), mean (SD)	1.5 (1.4)	1.4 (0.8)	0.03*		+
		Mortality, n (%)	11/43 (4.3)	11/398 (2.0)	0.21		ne
	Carr et al., 2018, USA [103]	Mortality (≥ 77 years old), OR (95% CI)	0.53 (0.3, 0.87)		NR	III-2	+
		Hospital LOS (≥ 78 years old), regression coefficient (95% CI)	− 0.55 (− 1.09, − 0.01		NR		+
	Fernandez et al., 2019, US [102]	ED LOS (minutes), mean (SD)	451.5 (376.1)	364.6 (277.9)	< 0.01*	III-2	+
		Hospital LOS (days), median (SD)	5.2 (4.5)	4.5 (3.4)	< 0.001*		+
		Ventilator days, median (SD)	0.2 (1.2)	0.1 (1.0)	< 0.001*		+
		Time to physician evaluation (minutes), mean (SD)	61.7 (87.4)	42.2 (67.0)	< 0.01*		+
		Time to computed tomography (minutes), mean (SD)	212.9 (661.5)	161.3 (550.9)	< 0.01*		+
		Mortality, n (%)	28/749 (3.7%)	39/1,454 (2.7%)	0.15		ne
	Hammer et al., 2016, US [101]	ED LOS ≤ 2 h, n (%)	61 (4.8)	65 (6.5)	0.08	III-2	ne
		ED LOS > 2 h, n (%)	1,210 (95.2)	933 (93.5)	0.08		ne
		Mortality, n (%)	105 (8.3)	76 (7.6)	0.57		ne
	Pelaez et al., 2021, USA [96]	Time between ED arrival to provider at bed (minutes), median (IQR)	0 (0, 3)	7 (2, 11)	< 0.001*	III-2	+
		Provider to bedside within 30 min of arrival, n (%)	73/91 (80)	121/142 (85)	0.32		ne
		Time between arrival to INR result (minutes), median (IQR)	38 (33, 48)	57 (40, 76)	< 0.001*		+
		Time between arrival and CT report (minutes), median (IQR)	52 (39, 61)	57 (43, 82)	0.01*		-
		Time between CT report and reversal intervention (minutes), median (IQR)	49 (-12, 213)	118 (29, 165)	0.51		ne
		Time between ED arrival to ED discharge (minutes), median (IQR)	147 (105,198)	120 (89,153)	0.01*		+
		Time between ED arrival to hospital admissions (minutes), median (IQR)	108 (83,167)	179 (135,275),	< 0.001*		-
		ED disposition to home, n (%)	44/91 (48)	96/142 (68)	< 0.01*		+
		Admitted to hospital by trauma service, n (%)	36/91 (82)	12/142 (13)	< 0.001		+
		Sustained injury, n (%)	27/91 (30)	33/142 (23)	0.27		ne
		Received reversal intervention, n (%)	5/91 (6)	15/142 (11)	0.39		ne
		Mortality, n (%)	1/91 (1)	3/142 (2)	0.56		ne
	Rittenhouse et al., 2015, USA [95]	Time from ED arrival to international normalised ratio test (minutes), median (IQR)	80 (57, 113)	13 (6, 27)	< 0.001*	III-2	+
		Time from ED arrival to head CT, median (IQR)	65 (42, 97)	35 (26, 48)	< 0.001*		+
		Patients discharged from ED, n (%)	76/337 (22.6)	233/415 (56.1)	NR		+
		Time in ED (hours), median (IQR)	3.4 (2.5, 4.6)	2.6 (1.9, 3.4)	< 0.001*		-
		Patients admitted to hospital, n (%)	261/337 (77.4)	182/415 (43.9)	NR		+
		Time from ED arrival to definitive care (hours), median (IQR)	2.6 (1.9, 3.6)	2.3 (1.7, 3.6)	0.34		ne
		LOS (days), median (IQR)	5.0 (3.0, 8.1)	3.7 (1.8, 6.7)	< 0.001*		+
		Stable on discharge, n (%)	324/337 (96.1)	406/415 (97.8)	0.17		ne
		Discharged to hospice, n (%)	7/337 (2.1)	5/415 (1.2)	0.34		ne
		Mortality, n (%)	6/337 (1.8)	4/415 (1.0)	0.33		ne
	Travers et al., 2021, USA [94]	Time from patient arrival in the ED to CT (hours), mean (SD)	2.4 (0.7)	0.6 (0.4)	< 0.001*	III-2	+
		ED LOS (hours), mean (SD)	4.7 (1.9)	2.6 (1.4)	< 0.001*		+
		Hospital LOS (days), mean (SD)	6.3 (4.5)	5.0 (4.4)	0.36		ne
		Mortality, n (%)	0 (0.0)	2 (3.6%)	0.94		ne
	van der Zwaard et al., 2020, The Netherlands [98]	Decided not to undergo surgery, n (%)	5/185 (2.7)	18/ 197 (9.1)	< 0.01*	III-2	+
	Wallace et al., 2019, US [97]	ED LOS (hours), mean (SD)	6.8 (2.9)	3.8 (2.4)	< 0.001*	III-2	+
		Hospital LOS (days), mean (SD)	7.4 (6.7)	5.0 (3.5)	< 0.01*		+
		Complications, n (%)	24/80 (30)	19/191 (10)	< 0.001*		+
		Mortality, n (%)	5 (6.3)	9 (4.7)	0.6		ne
	Wiles et al., 2018, US [100]	ED LOS, hours	5.8	4.5	< 0.01*	III-2	+
		Hospital LOS, days	4.4	4.8	0.02*		-
		Time from ED to admission to operating room	NR	NR	0.1		ne
		Hospital admission, %	98.4	61.9	NR		+
		Admission to skilled nursing facility/ inpatient rehabilitation, %	76.7	18.4	NR		+
		Mortality, %	1.6	4.8	NR		-
		Complications, %	16.4	1.6	< 0.01*		+
	Wright et al., 2014, UK [99]	Same-day discharge, OR (95% CI)	1.4 (1.2, 1.6)		< 0.001*	III-2	+
		Hospital LOS reduction, % (days)	18.2 (1.7)		< 0.001*		+
	Keyes et al., 2019, USA [105]	Diagnosed with intracranial haemorrhage on initial CT, n (%)		35/38 (92.1)		III-3	+
		Diagnosed with intracranial haemorrhage on repeat CT, n (%)		3/38 (0.8)			+
		Anticoagulation reversal protocol ordered, n (%)		29/38 (76.3)			
		Arrival to anticoagulation reversal protocol (minutes), mean (SD)		67.4 (27.6)			

Characteristics of interventions and study populations reported in Supplement 1. CI: confidence interval. *CT* computed tomography, *ED* Emergency Department, *INS* international normalised ratio, *IQR* interquartile range, *LOS* length of stay, *ne* no effect, *NR* not reported, *SD* standard deviation, + positive effect, *-*, negative effect

*denotes statistical significance

### Comprehensive assessment and multifaceted care

Three quasi-experimental studies evaluated screening and referral or multidisciplinary assessment interventions [48, 49, 53]. Compared to usual care, the Geriatric Emergency Room Innovations for Veterans intervention increased consults to pharmacy (43.4% vs 26.9%; *p* < 0.001) and social work (55.0% vs 18.2%; *p* < 0.001), and referrals to outpatient services (17.7% vs 5.8%; *p* < 0.001) and Home-Based Primary Care (30.4% vs 7.8%; *p* < 0.001) [53]. Lower rates of hospital admission (50.1% vs 57.5%; *p* < 0.01) and 30-day hospital readmission (56.8% vs 64%; *p* < 0.001) were also noted. In another, risk screening and interventional care planning had no effect on LOS, hospital admission or 30-day ED representation [49].

Instead of implementing an ED-based specialist geriatric team, one program integrated existing hospital consultants with geriatric training into the existing ED observation unit and introduced unit protocols to guide comprehensive assessment and multidisciplinary referral for non-admitted patients [48]. Following implementation of this program, 89 (40.3%) patients received at least one consultation. The most common protocol used was for transient ischaemic attack, but the use of this protocol (19.1%) was similar to patients who did not receive comprehensive assessment (18.1%). There was no effect on hospital admission or LOS in observation unit.

Older Person Technical Assistants (OPTAs) were introduced in an ED to conduct multifactorial screening (including cognition, delirium, falls risk, pain, pressure injury, nutrition and caregiver strain) and inform assessment and care planning for older adults (≥ 75 years) [52]. The OPTAs increased the completion of screening of cognition from 1.5% to 38% (*p* < 0.001) and review of pain from 29 to 75% (*p* < 0.001), attaining similar screening scores to the Aged Services Emergency Team Registered Nurses; supportive care, such as giving food or fluids, orientation, toileting, mobilisation, and pressure care, also significantly improved (*p* < 0.001) [52].

Two pre-post studies implemented Geriatric Emergency Department Intervention (GEDI), a nurse-led intervention to improve health outcomes for frail older adults in ED. Though the primary aim of GEDI is better patient care, both studies predominantly reported system performance measures [47, 50]. In one study, GEDI was associated with a small increase in hospital LOS [0.63 days] and a lower risk of in‑hospital death at hospital A, and a small decrease in hospital LOS (0.12 days) with no change in in‑hospital death at hospital B [47]. In the other study, GEDI increased likelihood of discharge, reduced ED LOS, had no effect on hospital LOS, risk of death or 28-day ED representation [50]. Six studies described comprehensive older adult assessment programs in ED primarily to reduce hospital admission, four of which reported reducing avoidable hospital admissions [31, 35, 36, 56]; of these, one study was associated with increased mean hospital LOS [36], and two showed no effect on reducing ED re-attendance [31, 35].

Two further studies investigated the impact of a validated clinical tool to screen older adults in the ED at high risk of prolonged ED LOS and hospitalisation [39, 40]. The tool provided geriatric recommendations customised to improve ED care for those identified as high risk. The first study analysed outcomes for patients visiting ED on a stretcher, and found no effect on hospital admission, but reduced hospital LOS for intervention participants admitted to hospital (β =  − 2.07, 95% CI: − 3.67 to − 0.47) [39]. The second study analysed outcomes of the same intervention for those presenting with neurocognitive disorders and found these patients less likely to be admitted to hospital than the control group (OR ≤ 0.61, 95% CI: 0.40 to 0.93) [40]. However, both cohorts had a longer LOS in ED [39, 40]. In contrast, a Geriatric Emergency Medicine Unit for managing neurocognitive disorders in older patients was associated with increased hospital admission in the intervention group compared to usual care [37]. Nevertheless, the patients treated by the unit were less likely to be readmitted within 30 days than patients receiving usual care (OR: 0.65; 95% CI: 0.46 to 0.94; *p* = 0.02).

One study of a Comprehensive Geriatric Assessment (CGA)-based nurse-led model of care in the ED found reduced ED LOS compared to usual care (median 12.7 h vs 19.1 h, *p* < 0.001) [32], while another lengthened ED LOS (6.4 h vs 5.3 h; *p* < 0.001) [42]. Both studies measured future hospitalisations to assess the effectiveness of the interventions for older adults at high-risk of hospitalisation. The first reported increased hospital admission compared to usual care (70% vs 67%, *p* < 0.01) [32], while another study using a care coordination team increased 28-day ED re-attendance (14.8% vs 17.9%, *p* = 0.05) and one-year unplanned hospital admissions (29.5% vs 43.4%, *p* < 0.001) [32, 41]. However, in both studies those not assessed as high risk of hospitalisation were used as the usual care comparators [32, 41].

### Minimising functional decline

Intervention specifically to minimise functional decline included patient outcome measures of function and patient experience through self-reported quality of life. A two-stage screening and nursing assessment intervention for older patients in the ED who were at increased risk of functional decline was evaluated in three RCTs [62, 63, 83]. The intervention significantly reduced functional decline in one RCT (OR: 0.5; 95%CI: 0.3 to 0.9) [83], but did not affect 4-month decline in functional status or death in another RCT [62]. Intervention participants were more likely to have documented referrals to their primary physician (OR: 1.9; 95% CI: 1.0 to 3.4), but many did not contact or visit their physician as a result of the referral (OR: 1.2; 95% CI: 0.7 to 2.3) [63]. Intervention participants were more likely to re-present to the ED within 30 days (OR: 1.6; 95% CI: 1.0 to 2.6) [63]. Three quasi-experimental studies explored interventions to attenuate functional decline [66, 77, 78] with mixed results over different measures. Older people who didn’t receive an intervention comprising review by an Advanced Practice Nurse followed by multidisciplinary geriatric assessment and follow-up care when discharged had a higher rate of progression to a poorer frailty category at 1, 3, and 6 months (*p* < 0.05) compared to those that did receive the intervention. However, there were no differences in ED re-attendance, hospital admission or mortality between the intervention and non-intervention group [66]. Older people receiving a multicomponent frailty intervention comprising CGA, frailty education, and a discharge transition package were more likely to maintain/improve independence in performing Activities of Daily Living (ADL) at 12 months and had lower ED re-attendance at 6 months (rate ratio: 0.35; 95% CI: 0.13 to 0.90; *p* = 0.03) compared to usual care [78]. A risk stratification followed by rapid geriatric screening intervention had significant preservation in function to perform ADL (Modified Barthel Index Score (MBI): − 0.99 vs − 0.24; *p* < 0.01; ADL: − 2.57 vs + 0.45; *p* < 0.01) at 12 months compared to usual care [77]. There were no significant reductions in ED re-attendance and hospital admission between study groups.

Other interventions included geriatric assessment in an ED Observation Unit which identified unmet needs in 32 patients (10.2%) who would have otherwise been discharged. The study reported reduced 3-month ED re-attendance (IRR: 0.58; 95% CI: 0.42 to 0.81) and 3-month hospital admissions (IRR: 0.61; 95% CI: 0.41 to 0.92) compared to usual care [76].

Another cohort study explored the provision of physical therapy services in the ED for older adults who fall and found patients receiving physical therapy were less likely to represent to the ED within 30- or 60-days (OR: 0.7; *p* < 0.001) [79].

### Managing falls risk

Two RCTs investigated the effects of a multidisciplinary team intervention for older adults who sought care in the ED after having a fall [33, 34]. The interventions had no significant effect on ED LOS [34], discharge destination [34], or hospital admissions [33, 34], but some participants were less likely to experience subsequent fall-related ED visits (IRR: 0.34; 95% CI: 0.15 to 0.76) or all-cause ED visits (IRR: 0.47; 95% CI 0.29 to 0.74) within 6 months compared to control participants [33].

The predominant measure of effectiveness of interventions in ED to manage patients falls risk in four studies included further falls, and repeated ED presentations or hospitalisations with fall-related injury [41, 70, 71, 81]. One of the four studies, which adopted a standardised and systematic pathway for patients presenting to an ED after a fall [41], was associated with a higher rate of ED discharge (66% post vs 46% pre; *p* = 0.001), shorter ED LOS (3.6 h post vs 6.5 h pre; *p* < 0.001) and hospital LOS (2 days post vs 6 days pre; *p* < 0.001).

### Palliative or supportive care

Patient outcomes, patient satisfaction, and system performance measures were reported in studies of interventions for supportive or palliative care for older adults in the ED. An Advanced Illness Management program in the ED was adopted/ implemented to better identify those with advanced illness and promote ED-led goals of care discussion and referrals to hospice from the ED [72]. A second study reported outcomes from introduction of a Geriatric and Palliative-ED partnership. The partnership was reported to have achieved high patient satisfaction, and while there was no significant change in 30-day ED revisit, the number of hospital admissions at 30-day ED revisit was reduced (40% post vs 57% pre; *p* = 0.01) [85].

One RCT found delivering dietetic assessment, nutrition intervention and follow-up to older adults in ED had no significant impact on weight change, hospital LOS, quality of life, depression, or further decline in malnutrition status for participants receiving individualised dietary counselling compared to participants receiving usual care [51].

### Assessment and management of pain

Two studies targeted pain management, measuring system performance, patient outcomes, and patient experience [74, 80]. In both studies, staff education significantly improved pain management of older adults in ED. One study demonstrated more regular pain assessment and reduction in pain [80]; the authors were also able to describe patient experience by using a subjective pain scale rather than a quantitative score only. Another pre-post study showed staff education subsequently increased use of nerve blocks as an evidence-based mode of analgesia for elderly patients with a fractured neck of femur in the ED [74].

### Staff education

System performance measures were used to measure the impact of educating nursing staff in comprehensive care for older adults in the ED to improve screening for depression and altered mental status [73], knowledge of geriatric concepts and use of geriatric assessment tools [88].

### Medication safety

Five studies targeted safer medication practice [89–93], measuring system performance outcomes, including the prescription of potentially inappropriate medications (PIMs) or Fit for the Aged (FORTA), and polypharmacy. Two pre-post studies evaluated a program (EQUiPPED) combining education, electronic health record based clinical decision support tools, and individual provider audit and feedback with peer benchmarking [89, 90]. One implemented EQUiPPED at four sites and found significant reductions in the prescribing of PIMs at all four sites (mean reduction from 1.7%; *p* = 0.04 to 6.8%; *p* < 0.001) [89]. The other pre-post study implemented EQUiPPED at three sites and found a minor but significant reduction in PIMs after implementation at one site [0.5%; *p* = 0.02] [90]. However, no significant reductions in PIMs were found after implementation at the other two sites.

A pre-post study appraised a computer-based and pharmacist-assisted medication review initiated in the ED that reduced major polypharmacy [≥ 10 medications] and PIMs at hospital discharge [91].

Junior Medical Officers were less likely to prescribe a PIM after education [93] and PIMs were also significantly reduced following introduction of telemedical geriatric assessment [92].

### Geriatric trauma protocol

Trauma protocols specific to geriatric patients were introduced to reduce mortality in patients older than 65 years compared with younger patients with similar injury [99–104]. Strategies to capture geriatric patients included widening existing trauma activation alerts, introducing a new triage tier, and implementing a specific geriatric trauma team. Patient outcomes including mortality and morbidity were measured and system performance indicators such as the number of patients included in trauma activation, time to be seen, time to treatment, LOS and patient disposition, were collected. Widening capture of older patients increased existing trauma team workload, but did not always result in better outcomes [99, 102, 104], whereas introduction of a third-tier trauma protocol reduced ED LOS (5.5 h pre vs 4.5 h post; *p* < 0.01), decreased hospital admissions (98.4% pre vs 61.9% post), and lowered complication rates (16.4% pre vs 1.6% post; *p* < 0.01) in one study [100]. However, hospital LOS increased (4.4 days pre vs 4.8 days post; *p* = 0.02), as did mortality (1.6% pre vs 4.8% post). The establishment of a Triage and Rapid Elderly Assessment Team increased same-day discharges (OR 1.4; 95% CI: 1.2 to 1.6; *p* < 0.001) and reduced mean hospital LOS by 1.8 days (*p* < 0.001) compared to the pre-establishment period [99].

### Management of anticoagulated older adult with head injury

Three studies specifically targeted anticoagulated older adults with head injury, measuring system performance, including time to be seen, time to treatment, LOS, and patient disposition [94–96]. All three studies reported faster completion of investigations (CT scan and International normalised ratio (INR) test).

### Assessment and management of hip fracture

Two studies specifically targeted hip fracture, one measuring system performance, the other measuring patient outcomes [97, 98]. A pre-post study evaluated the effects of a multidisciplinary hip fracture care pathway for the care of elderly patients and found the pathway was associated with reduced ED LOS (3.8 h vs 6.8 h pre; *p* < 0.001), hospital LOS (5 h vs 7.4 h pre; < 0.01) and complications (10% vs 30% pre; < 0.001) [97]. A quasi-experimental study of older patients with hip fracture compared patients who received pre-operative CGA with shared decision making by a geriatrician to usual care. More patients who received the intervention opted for non-surgical management, compared to usual care (9.1% vs 2.1%; *p* < 0.01) [98].

## Discussion

**Fig. 2:**
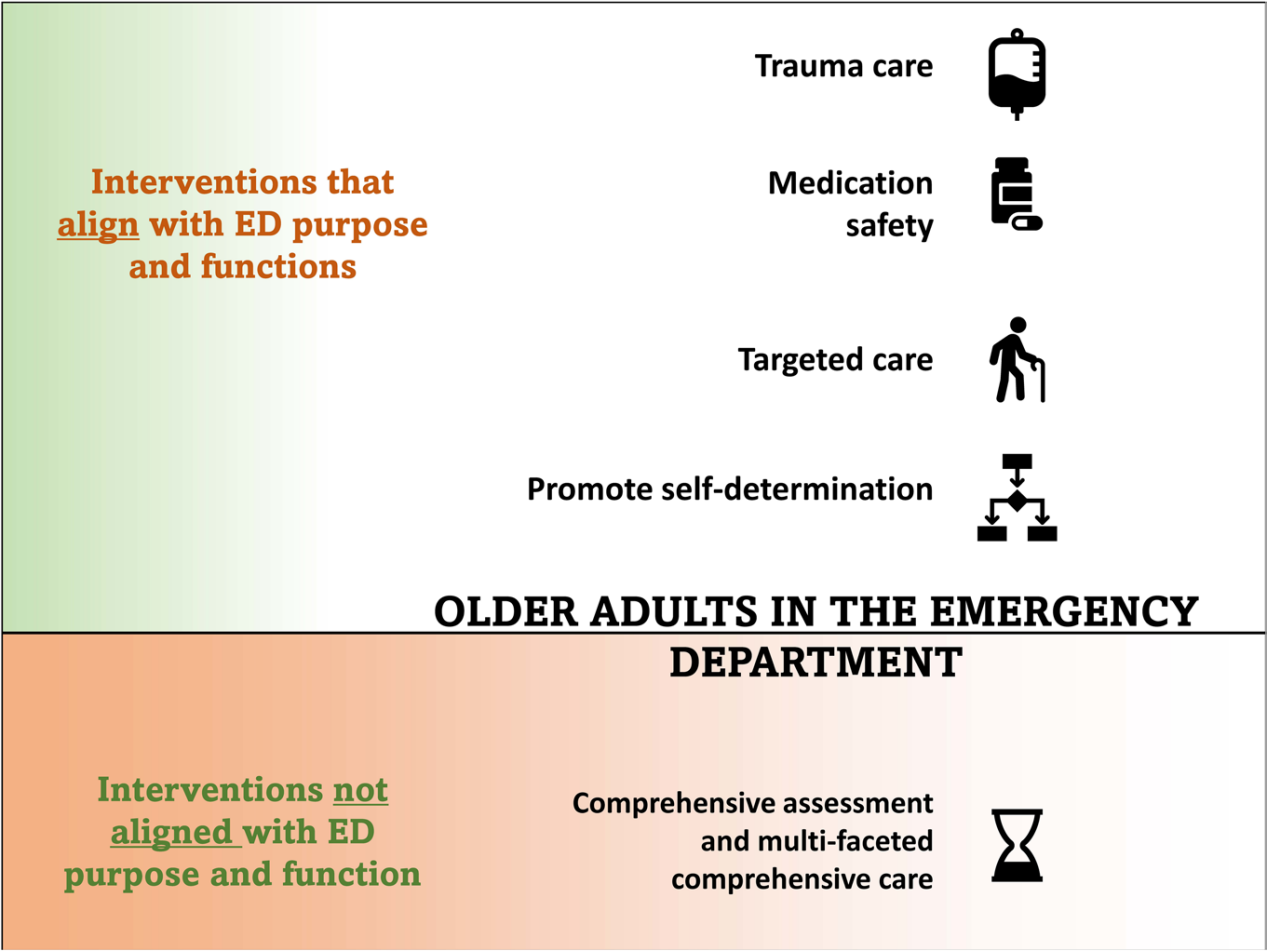
ED interventions for older adults

We examined the peer-reviewed literature for strategies used to improve value-based healthcare delivery for older adults in ED. Whereas some of the comprehensive assessment and multifaceted interventions reduced avoidable hospital admissions, most of those identified in the current review increased the time older adults spent in ED by increasing the depth of care provided and did not reduce ED representations or further hospitalisations. There is a misalignment between such comprehensive care delivered in ED for older adults and ED performance measures oriented to rapid assessment and referral. In contrast, targeted interventions such as those to reduce polypharmacy, or respond to acute trauma in older adults were found to align with ED function and ED performance measures and show promise as more effective ED interventions for older adults (Fig. 2). Critically, there were few measures used to understand the impact of strategies on patient experience and even fewer that considered provider experience.

Despite the quadruple aim of delivering care that improves health outcomes that matter to patients, improving the experiences of receiving and of providing care, and improving the effectiveness and efficiency of care, the current review highlights that the experiences of patients and staff are not routinely captured. EDs are purpose-built to provide 24-h access to urgent care and a pathway to hospital and community healthcare services [2, 106, 107]. System performance measures are oriented to, and may financially reward, rapid general assessment and urgent care delivery [2]. Amid global workforce pressures and shortages contributing to burnout and attrition [12], it is crucial to improve workforce experiences in implementing care improvement strategies for older adults that are congruent with ED function and performance measures.

The multi-faceted nature of interventions, complex patient variables, and mixed results amongst the included studies made it difficult to identify what components of comprehensive care in ED are most effective. Delivering multi-faceted comprehensive care for older adults in a time-pressured ED environment is challenging. ED system performance incorporates measures such as number of patients seen, waiting time, and their LOS [107]. These are valid measures in a care space where care demand is unlimited and continued function depends on adequate patient flow. The ED environment is not designed for extended patient stays – there is little differentiation between night and day, little privacy, fewer facilities for toileting and bathing, and excessive noise levels [108]. ED staff are specifically trained and organised to promote rapid assessment and referral. This means that strategies aiming to provide care beyond the scope of the ED purpose may compromise ED system functioning and may inadvertently contribute to worse patient outcomes, patient and staff experience [107]. Older adults often present to ED with multiple comorbidities, multiple medications, and declining function that warrant careful assessment and management alongside their presenting complaint [14, 109]. Older adults are a high-risk population and may need multi-faceted care, but an alternative to the ED environment for prolonged comprehensive assessment and care is warranted. Alternatives may include strengthening community care or dedicated older adult EDs. Transitioning older adults more quickly to a hospital environment that better meets their needs might be possible with low acuity units to accommodate those patients ready for discharge and these may be a lower cost option.

Strategies for managing older adult trauma and medication safety were better aligned with ED purpose and provided better outcomes in ED for older adults. Notable among the strategies for medication safety was lower cost intervention to educate junior medical staff about good prescribing practice, as well as higher cost interventions such as pharmacist and geriatric telemedical review. The latter may be unattainable in some EDs, but the range of interventions demonstrates low-resource actionable strategies are possible and can be effective. Another strategy might include patient education to assist them to advocate against polypharmacy or PIMs for themselves as interventions in this review that promoted self-determination reported favourable patient experience measures.

Favourable patient experience was reported with interventions to better manage pain, and interventions to identify advanced illness to prompt goals of care discussions [72, 80, 98]. Gathering patient experience in ED is difficult given exigency and distress inherent in this care context. A novel approach was provided by Hogan et al. [80] who transformed the quantitative pain management scale to a qualitative comfort scale. An example of a proxy measure was the selection of an alternative non-surgical pathway for hip fracture [98]. More consistent reporting of outcome measures, such as those advanced by the International Consortium for Health Outcomes Measurement [110], may assist in better identifying replicable high impact interventions. Overall, few interventions measured staff experience. This may be because interventions that improve the ED working environment are scarce [111], so these measures are underdeveloped. It is known that ED staff are negatively impacted by high levels of occupational stress and burnout [12, 112], which in turn negatively impacts ED performance [113] and patient safety [114]. Improvement initiatives codesigned with patients and providers may be helpful in ensuring change is high-value, appropriate, prioritised and sustained, providing opportunities for front line clinicians to reconnect with the values that motivated them to work in the sector [18]. The alternative of top down initiatives can introduce more complexity for frontline staff with little or no benefit [18, 21].

## Limitations

The current review identified a wide range of complex interventions implemented in a variety of ED settings. ED interventions interact with the characteristics, circumstances, and unique factors of the ED where they are implemented [115]. Where an intervention was associated with favourable outcomes, contextual factors may have influenced these outcomes, but these were not consistently described across studies. The nature of pragmatic naturalistic study designs may introduce bias: allocation concealment was not used in 4/9 RCT studies, and blinding did not occur/was not possible in most studies; most studies were quasi-experimental/non-randomised studies – participants in comparisons were not always similar or it was unclear if participants were similar in 25/49 (just over 50%) of studies. Consequently, it was not possible to identify the key elements of interventions and features of ED environments that influence outcomes. Consistent reporting of interventions using reporting guidelines, such as the Template for Intervention Description and Replication (TIDieR) checklist [116], would be helpful in future research and for the overall development of the field.

We also made pragmatic decisions to manage the vast literature base on older adults in the ED and to focus on the aims of the review. We eliminated abstracts that only reported screening but no subsequent intervention in ED or outcomes of interest, and those where the intervention was delivered outside of ED e.g., general ward-based care or community care. Some articles addressed specific illness such as Chronic Obstructive Pulmonary Disease or Stroke. Even though chronic illness is prevalent in older adults, these articles were omitted from the review if the mean age of participants was < 65 or not reported. This review included only articles published in the peer-reviewed literature which may also have excluded relevant, but unpublished material. Additionally, only interventions published in the English language were included in this study; this is a limitation to the external validity, as studies in languages other than English are likely to be valuable in this area.

## Conclusion

Strategies identified to improve ED care for older adults included comprehensive care, recognition and response to acute deterioration, and medication safety. Few studies reported on all aspects of the quadruple aim and no intervention demonstrated improved ED care delivery across all four domains. Future interventions should better embed patient experience and be inclusive of staff experience; patient and provider input at the design stage may advance prioritisation of higher-impact interventions aligned with the function of the system and the pace of change. More consistent evaluation and reporting to illuminate contextual factors would support replication and wider adoption of promising high value intervention. It is crucial that future strategies to improve care delivery in ED align the needs and priorities of older adults and with the purpose of the ED system to assure sustainable improvement effort and critical functioning of the ED as an interdependent component of the health system.

### Supplementary Information

**Additional file 1: Supplement 1.** Search strategy.

**Additional file 2: Supplement 2.** Characteristics of intervention studies for older adults in the ED.

**Additional file 3: Supplement 3.** JBI Critical Appraisal Checklists for included studies.

## Data Availability

Data is available from the corresponding author upon reasonable request.
