# Correction: Strategies to improve care for older adults who present to the emergency department: a systematic review

**DOI:** 10.1186/s12913-024-10766-x

**Published:** 2024-03-07

**Authors:** Luke Testa, Lieke Richardson, Colleen Cheek, Theresa Hensel, Elizabeth Austin, Mariam Safi, Natália Ransolin, Ann Carrigan, Janet Long, Karen Hutchinson, Magali Goirand, Mia Bierbaum, Felicity Bleckly, Peter Hibbert, Kate Churruca, Robyn Clay-Williams

**Affiliations:** 1 Australian Institute of Health Innovation, Macquarie University, Level 6, 75 Talavera Road, North Ryde, 2109 Australia; 2 Institute of Medical Sociology, Health Services Research and Rehabilitation Science (IMVR), University of Cologne, Cologne, Germany; 3 Internal Medicine Research Unit, University Hospital of Southern Denmark, Aabenraa, Denmark; 4 Department of Regional Health Research, University of Southern Denmark, Odense, Denmark; 5 Universidade Federal Do Rio Grande Do Sul, Porto Alegre, RS Brasil; 6 Allied Health and Human Performance, IIMPACT in Health, University of South Australia, Adelaide, 5001 Australia

**Correction to**: *BMC Health Services Research* (2024) 24:123. 10.1186/s12913-024-10576-1.

In this article, the author name Felicity Bleckly was incorrectly written as Felicity Bleckley. The author group has been updated above and the original article has been corrected.

