# Supplementary material for: Strategies to improve care for older adults who present to the emergency department: a systematic review
Source: BMC Health Serv Res. 2024 Feb 8;24:178. doi: 10.1186/s12913-024-10576-1 (PMC10851482; doi:10.1186/s12913-024-10576-1)
Supplement: Supplementary file 1 — Additional file 1: Supplement 1. Search strategy. [file 12913_2024_10576_MOESM1_ESM.docx]

**Supplement 1 – Search strategy**

Contents

[1. Elsevier Scopus 2](#_Toc111718515)

[2. Ovid Embase 4](#_Toc111718516)

[3. EBSCOhost CINAHL Complete 6](#_Toc111718517)

[4. Ovid MEDLINE Complete 8](#_Toc111718518)

# Elsevier Scopus

| **SUBJECT #1: Emergency Department**  **KEYWORDS:** ( TITLE-ABS-KEY ( ( "hospital emergency service*" OR "emergency department*" OR "emergency unit*" OR "emergency ward*" OR "emergency room" * ) ) )  **MESH/ SUBJECT HEADING :** not applicable for Elsevier Scopus |
| --- |
| **SUBJECT #2: Improvement**  **KEYWORDS** ( TITLE-ABS-KEY ( "quality improve*" OR "quality performance" OR "quality intervention" OR "care improve*" OR "quality assess*" OR "quality assurance" OR "geriatric assessment" OR "nursing assessment" OR "case management" OR "patient care planning" OR "discharge planning" OR "multidisciplinary intervention" OR "improv* outcomes") )  **MESH/ SUBJECT HEADING :** not applicable for Elsevier Scopus |
| **SUBJECT #3 Outcomes**  **Keywords:** TITLE-ABS-KEY ( "Clinical Outcome*" OR ( patient W/2 outcome* ) OR ( consumer W/2 outcome* ) OR "health outcomes" OR reattendance OR representation OR readmission OR "re-attendance" OR "adverse events" OR "clinical errors" OR "medication error" OR "missed diagnosis" OR "mortality" OR "morbidity" OR "pain" OR "quality of life" OR "HRQoL" OR "health related quality of Life" OR "patient satisfaction" OR "patient experience" OR ( patient W/2 ( experience OR perspective OR opinion OR perception OR view OR feedback OR preference ) ) OR "staff experience" OR "job satisfaction" OR "intention to stay" OR "work environment" OR "employee complaint ratio" OR "patient wait time satisfaction" OR "patient reported problems" OR "patient reported outcome" OR "patient reported experience" OR "patient complaints" OR "left before treatment complete" OR "LAMA" OR "left against medical advice" OR "LWBS" OR "left without being seen" OR "LBTC" OR "left without Treatment complete" OR "PWLBST" OR "Patients who left before supposed to" OR "PREM" OR "patient reported experience measure" OR "ED performance" OR "performance indicator" OR "performance measure" OR "benchmark" OR "length of stay" OR los OR "functional decline" OR "hospital admission" OR "admission rates" OR hospitalization OR hospitalisation OR recidivism OR "service use rates" OR "health care quality" OR "satisfaction with care" OR "caregiver outcome" )  **MESH/ SUBJECT HEADING :** not applicable for Elsevier Scopus |
| **SUBJECT #4: Older Adults**  **KEYWORDS:** TITLE-ABS-KEY ( ( "older adult" ) OR ( "older people" ) OR ( "older person" ) OR ( aged ) OR ( elder* ) OR ( geriatric* ) OR ( gerontol* ) OR ( senior* ) OR ( aging ) OR ( ageing ) OR ( "over 65" ) OR ( "age 65" ) OR ( "old age" ) OR ( 65 years ) OR ( "senior citizen" ) OR ( senium ) OR ( "aged hospital patient" ) OR ( palliat* ) AND NOT "middle aged")  **MESH/ SUBJECT HEADING :** not applicable for Elsevier Scopus |
| **LIMITATIONS:**  AND NOT ( TITLE-ABS-KEY ( pediatric OR child* OR adolescent OR ambulatory OR outpatient) |

#

# Ovid Embase

| **SUBJECT #1: Emergency Department**  **KEYWORDS:** ("hospital emergency service*" or "hospital emergency service*" or "emergency department*" or "emergency unit" or "emergency ward*" or "emergency room*").ti,ab  **MESH/ SUBJECT HEADING :** emergency ward/ or hospital emergency service/ or emergency health service/ |
| --- |
| **SUBJECT #2: Improvement**  **KEYWORDS** ("quality improve*" OR "quality performance" OR "quality intervention" OR "care improve*" OR "quality assess*" OR "quality assurance" OR "geriatric assessment" OR "nursing assessment" OR "case management" OR "patient care planning" OR "discharge planning" OR "multidisciplinary intervention" OR "improv* outcomes").ti,ab  **MESH/ SUBJECT HEADING :** total quality management/ or Geriatric Assessment/ or Case Management/ or Nursing Assessment/ or health risk assessment/ |
| **SUBJECT #3 Outcomes**  **Keywords:** ( "Clinical Outcome*" OR ( patient AND outcome ) OR ( consumer AND outcome ) OR "health outcomes" OR reattendance OR representation OR readmission OR "re-attendance" OR "adverse events" OR "clinical errors" OR "medication error" OR "missed diagnosis" OR "mortality" OR "morbidity" OR "pain" OR "quality of life" OR "HRQoL" OR "health related quality of Life" OR "patient satisfaction" OR "patient experience" OR ( patient ADJ2 ( experience OR perspective OR opinion OR perception OR view OR feedback OR preference ) ) OR "staff experience" "staff experience" OR "job satisfaction" OR "intention to stay" OR "work environment" OR "employee complaint ratio" OR "patient wait time satisfaction" OR "patient reported problems" OR "patient reported outcome" OR "patient reported experience" OR "patient complaints" OR "left before treatment complete" OR "LAMA" OR "left against medical advice" OR "LWBS" OR "left without being seen" OR "LBTC" OR "left without Treatment complete" OR "PWLBST" OR "Patients who left before supposed to" OR "PREM" OR "patient reported experience measure" OR "ED performance" OR "performance indicator" OR "performance measure" OR "benchmark"OR "length of stay" OR LOS OR "functional decline" OR "hospital admission" OR "admission rates" OR hospitalization OR hospitalisation OR recidivism OR "service use rates" OR "health care quality" OR "satisfaction with care" OR "caregiver outcome").ti,ab  **MESH/ SUBJECT HEADING :** treatment outcome/ or adverse outcome/ or hospital readmission/ or medical error/ or mortality / or morbidity/or pain/ or quality of life/ or patient preference/ or patient-reported outcome/ or performance indicator/ or health care quality/ or benchmarking/ or patient satisfaction/ or daily life activity/ or outcome assessment/ or patient referral/ or consultation/ or hospital admission/ or "length of stay"/ |
| **SUBJECT #4: Older Adults**  **KEYWORDS:** (("older adult*" or "older people" or "older person" or aged or elder* or geratric* or gerontol* or senior* or ageing or aging or "over 65" or "over 65" or "age 65" or "old age" or "senior citizen" or senium or "aged hospital patient").ti,ab) not ("middle aged").ti,ab  **MESH/ SUBJECT HEADING :** exp aged/ or exp palliative therapy/ |
| **LIMITATONS:**  not (pediatric or child*).ti,ab |

# EBSCOhost CINAHL Complete

| **SUBJECT #1: Emergency Department**  **KEYWORDS:**  TI ( "emergency service*" OR "hospital emergency service*" OR "emergency department*" OR "emergency unit" OR "emergency ward*" OR "emergency room*" )  OR AB ( "emergency service*" OR "hospital emergency service*" OR "emergency department*" OR "emergency unit" OR "emergency ward*" OR "emergency room*" ) **MESH/ SUBJECT HEADING :** MH ("Emergency Medical Services+"OR "Emergency Service+” OR "Acute Care+") |
| --- |
| **SUBJECT #2: Improvement**  **KEYWORDS**  TI TI ( "quality improve*" OR "quality performance" OR "quality intervention" OR "care improve*" OR "quality assess*" OR "quality assurance" ) OR AB ( "quality improve*" OR "quality performance" OR "quality intervention" OR "care improve*" OR "quality assess*" OR "quality assurance" OR "geriatric assessment" OR "nursing assessment" OR "case management" OR "patient care planning" OR "discharge planning" OR "multidisciplinary intervention" OR "improv* outcomes" )  **MESH/ SUBJECT HEADING :** (MH "Quality Improvement+") OR (MH "Evaluation and Quality Improvement Program") OR (MH "Quality Management, Organizational") OR (MH "Geriatric Assessment+") OR (MH "Geriatric Functional Assessment") OR (MH "Case Management") OR (MH "Nursing Assessment") OR (MH "Risk Assessment") |
| **SUBJECT #3 Outcomes**  **Keywords:** TI ( ( "Clinical Outcome*" OR ( patient N2 outcome ) OR ( consumer N2 outcome ) OR "health outcomes" OR reattendance OR representation OR readmission OR "re-attendance" OR "adverse events" OR "clinical errors" OR "medication error" OR "missed diagnosis" OR "mortality" OR "morbidity" OR "pain" OR "PROM*" OR "quality of life" OR "HRQoL" OR "health related quality of Life" OR "patient satisfaction" OR "patient experience" OR ( patient N2 ( experience OR perspective OR opinion OR perception OR view OR feedback OR preference ) ) OR "staff experience" OR "patient wait time satisfaction" OR "patient reported problems" OR "patient reported outcome" OR "patient reported experience" OR "patient complaints" OR "left before treatment complete" OR "LAMA" OR "left against medical advice" OR "LWBS" OR "left without being seen" OR "LBTC" OR "left without Treatment complete" OR "PWLBST" OR "Patients who left before supposed to" OR "PREM" OR "patient reported experience measure" OR "work environment" OR "employee complaint ratio" OR "ED performance" OR "performance indicator" OR "performance measure" OR "benchmark" OR "length of stay" OR LOS OR "functional decline" OR "hospital admission" OR "admission rates" OR hospitalization OR hospitalisation OR recidivism OR "service use rates" OR "health care quality" OR "satisfaction with care" OR "caregiver outcome" )  OR AB ( ( "Clinical Outcome*" OR ( patient N2 outcome ) OR ( consumer N2 outcome ) OR "health outcomes" OR reattendance OR representation OR readmission OR "re-attendance" OR "adverse events" OR "clinical errors" OR "medication error" OR "missed diagnosis" OR "mortality" OR "morbidity" OR "pain" OR "quality of life" OR "HRQoL" OR "health related quality of Life" OR "patient satisfaction" OR "patient experience" OR ( patient N2 ( experience OR perspective OR opinion OR perception OR view OR feedback OR preference ) ) OR "staff experience" OR "job satisfaction" OR "intention to stay" OR "work environment" OR "employee complaint ratio" OR "patient wait time satisfaction" OR "patient reported problems" OR "patient reported outcome" OR "patient reported experience" OR "patient complaints" OR "left before treatment complete" OR "LAMA" OR "left against medical advice" OR "LWBS" OR "left without being seen" OR "LBTC" OR "left without Treatment complete" OR "PWLBST" OR "Patients who left before supposed to" OR "PREM" OR "patient reported experience measure" OR "ED performance" OR "performance indicator" OR "performance measure" OR "benchmark" OR "length of stay" OR LOS OR "functional decline" OR "hospital admission" OR "admission rates" OR hospitalization OR hospitalisation OR recidivism OR "service use rates" OR "health care quality" OR "satisfaction with care" OR "caregiver outcome")  **MESH/ SUBJECT HEADING :** (MH "Treatment Outcomes+") OR (MH "Readmission") OR (MH "Health Care Errors") OR (MH "Mortality") OR (MH "Morbidity") OR (MH "Pain") OR (MH "PROM") OR (MH "Qualtiy of Life") OR (MH "Patient Preference") OR (MH "Patient-Reported Outcomes") OR (MH "Work environment") OR (MH "Quality of Health Care") OR (MH "Benchmarking") OR (MH "Process Assessment (Health Care)") OR (MH "Patient Satisfaction+") OR (MH "Activities of Daily Living+") OR (MH "Outcome Assessment") OR (MH "Patient Admission/SN") OR (MH "Length of Stay") OR (MH "Continuity of Patient Care+") |
| **SUBJECT #4: Older Adults**  **KEYWORDS:** TI ("older adult*" OR "older people" OR "older person" OR aged OR elder* OR geratric* OR gerontol* OR senior* OR aging OR ageing OR "over 65" OR "over 65" OR "age 65" OR "old age" OR "senior citizen" OR senium OR "aged hospital patient" )  OR AB ("older adult*" OR "older people" OR "older person" OR aged OR elder* OR geratric* OR gerontol* OR senior* OR aging OR ageing OR "over 65" OR "over 65" OR "age 65" OR "old age" OR "senior citizen" OR senium OR "aged hospital patient")  **MESH/ SUBJECT HEADING :** MH ("Hospitalization of Older Persons" OR "Health Services for Older Persons" OR "Gerontologic Care" OR "Aged, 80 and Over" OR “Palliative Care”) |
| **Filter:**  NOT ((TI (pediatric OR child*)) OR (AB (pediatric OR child*))) |

# Ovid MEDLINE Complete

| **SUBJECT #1: Emergency Department**  **KEYWORDS:** ("hospital emergency service*" or "hospital emergency service*" or "emergency department*" or "emergency unit" or "emergency ward*" or "emergency room*").ti,ab  **MESH/ SUBJECT HEADING :** Emergency Service, Hospital/ or Emergency Medical Service/ |
| --- |
| **SUBJECT #2: Improvement**  **KEYWORDS** ("quality improve*" OR "quality performance" OR "quality intervention" OR "care improve*" OR "quality assess*" OR "quality assurance" OR "geriatric assessment" OR "nursing assessment" OR "case management" OR "patient care planning" OR "discharge planning" OR "multidisciplinary intervention" OR "improv* outcomes").ti,ab  **MESH/ SUBJECT HEADING:** Quality Improvement/ OR Geriatric Assessment/ OR Case Management/ OR Nursing Assessment/ OR Risk Assessment/ |
| **SUBJECT #3 Outcomes**  **Keywords:** ( "Clinical Outcome*" OR ( patient AND outcome ) OR ( consumer AND outcome ) OR "health outcomes" OR reattendance OR representation OR readmission OR "re-attendance" OR "adverse events" OR "clinical errors" OR "medication error" OR "missed diagnosis" OR "mortality" OR "morbidity" OR "pain" OR "quality of life" OR "HRQoL" OR "health related quality of Life" OR "patient satisfaction" OR "patient experience" OR ( patient ADJ2 ( experience OR perspective OR opinion OR perception OR view OR feedback OR preference ) ) OR "staff experience" OR "job satisfaction" OR "intention to stay" OR "work environment" OR "employee complaint ratio" OR "patient wait time satisfaction" OR "patient reported problems" OR "patient reported outcome" OR "patient reported experience" OR "patient complaint*" OR "left before treatment complete" OR "LAMA" OR "left against medical advice" OR "LWBS" OR "left without being seen" OR "LBTC" OR "left without Treatment complete" OR "PWLBST" OR "Patients who left before supposed to" OR "PREM" OR "patient reported experience measure" OR "ED performance" OR "performance indicator" OR "performance measure" OR "benchmark" OR "patient satisfaction" OR "length of stay" OR "LOS" OR "functional decline" OR "hospital admission" OR "admission rates" OR hospitalization OR hospitalisation OR recidivism OR "service use rates" OR "health care quality" OR "satisfaction with care" OR "caregiver outcome").ti,ab  **MESH/ SUBJECT HEADING :** Treatment Outcome/ or Patient Reported Outcome Measures/ or "Patient Readmission"/ or Medical Errors/ or Mortality/ or Morbidity/ or Pain/ or "Quality of Life"/ or Patient Preference/ or Benchmarking/ or Patient Satisfaction/ or "Activities of Daily Living"/ or Outcome Assessment, Health Care/ or "Referral and Consultation"/ or Patient Admission/ or "Length of Stay"/ or "Continuity of Patient Care"/ |
| **SUBJECT #4: Older Adults**  **KEYWORDS:** ("older adult*" or "older people" or "older person" or aged or elder* or geratric* or gerontol* or senior* or ageing or aging or "over 65" or "over 65" or "age 65" or "old age" or "senior citizen" or senium or "aged hospital patient").ti,ab  **MESH/ SUBJECT HEADING :** frail elderly/ or aged/ OR Health Services for the Aged/ OR "Aged, 80 and over"/ OR Palliative Care/ |
| **LIMITATIONS:**  not ((pediatric or child* or ambulatory or outpatient).ti,ab or (Middle Aged/)) |
