# Supplementary material for: Strategies to improve care for older adults who present to the emergency department: a systematic review
Source: BMC Health Serv Res. 2024 Feb 8;24:178. doi: 10.1186/s12913-024-10576-1 (PMC10851482; doi:10.1186/s12913-024-10576-1)
Supplement: Supplementary file 2 — Additional file 2: Supplement 2. Characteristics of intervention studies for older adults in the ED. [file 12913_2024_10576_MOESM2_ESM.docx]

**Supplement 2 - Characteristics of intervention studies for older adults in the ED**

| **Author, Year,**  **Country** | **Quality indicator, problem** | **Study aim** | **Intervention** | **Participating**  **EDs (n)** | **Participants intervention (n)** | **Participants**  **Control (n)** | **Study design** | **Outcome measure** |
| --- | --- | --- | --- | --- | --- | --- | --- | --- |
| Ageron et al., 2016, France | Comprehensive care | To assess effectiveness of a healthcare intervention program for the management of elderly patients admitted to EDs after a fall | **Management of falls risk**  Information and training tools to improve patient management, e.g., protocols, posters, website, and smartphone app | 13 | 2,426 | 2,684 | Pre-post | System performance Patient outcomes |
| Aldeen et al., 2014, USA | Comprehensive care | To describe characteristics of patients that these GEDI nurse liaisons see, and measure the admission rate of these patients | **General assessment and multi-faceted care coordination to reduce avoidable hospital admissions and/or LOS and/or improve ED flow**  Geriatric Nurse Liaison Model. All older adults (≥65) entering the ED were screened using the ISAR tool. | 1 | 408 | 6,806 | Quasi-experimental | System performance Patient outcomes |
| Arendts et al., 2013, Australia | Comprehensive care | To compare important clinical outcomes (mortality, ED re-attendance and re-hospitalisation) in patients requiring facilitated discharge using an allied health care coordination team based in the ED to those not requiring allied health input | **General assessment and multi-faceted care coordination to reduce avoidable hospital admissions and/or LOS and/or improve ED flow**  Comprehensive allied health input from a care coordination team prior to discharge | 2 | 1,098 | 1,098 | Quasi-experimental | System performance Patient outcomes |
| Arendts et al., 2020, Australia | Comprehensive care | To: (1) design and implement a standardised and systematic approach to patients presenting to an ED after a fall; and (2) achieve hospital efficiency gains, such as reduced hospital length of stay, through implementation of this approach | **Management of falls risk**  Direct admission to an ED short stay unit, standardised assessment of cognition,  medications, mobility and discharge risk, and access in the ED to a geriatric consultation service for complex patients | 1 | 873 | 562 | Pre-post | System performance Patient outcomes  Staff experience |
| Argento et al., 2014, USA |  | To report on experiences using APRNs embedded in an established ED | **General assessment and multi-faceted care coordination to better address patient needs**  Two full-time equivalent APRNs who cover the ED seven days a week between the hours of 9AM-7PM. The APRNs are highly visible and carry a dedicated phone line to be reached by the ED staff or patient families. Patients > 65 are screened with the Triage Risk Screening Tool. For any patient over the age of 65 with a Triage Risk Screening Tool score of 2 or higher, the APRN contacts the ED physician or, for patients with private doctors in the community, calls the community doctor directly to request permission to perform an evaluation. The APRN performs a CGA focusing on disposition decision making, acuity of care, and goals of care | 1 | In FY 2012: 1,302 consults; In FY 2013: 1,660 consults | - | Cohort | System performance Patient outcomes Patient experiences |
| Basic et al., 2002a, Australia |  | To evaluate the ability of a nurse practitioner in geriatrics, working in the ED of a tertiary referral hospital, to assess high-risk elderly patients comprehensively. A secondary aim was to explore patient characteristics associated with referral to community aged care services | **General assessment and multi-faceted care coordination to attenuate functional decline**  Emergency department aged care liaison nurse - study patients were assessed by a NP experienced in multidimensional assessment and care of the elderly | 1 | 469 | - | Descriptive | System performance |
| Basic et al., 2005, Australia |  | To determine whether early geriatric assessment (in the form of an aged care nurse intervention based in the ED) reduced admission to the hospital, inpatient LOS, or functional decline during the hospitalisation. | **General assessment and multi-faceted care coordination to attenuate functional decline**  Early geriatric assessment by an aged care nurse and intervention based in the ED | 1 | 114 | 110 | RCT | System performance Patient outcomes |
| Beauchet et al., 2021, Canada |  | To examine whether the use of ER2 tool in daily ED practice reduce the length of stay in ED and hospital, and hospitalisation in older adults visiting ED on stretcher. | **General assessment and multi-faceted care coordination to reduce avoidable hospital admissions and/or LOS and/or improve ED flow**  ER2 is a validated clinical tool which screens older adults visiting ED at high risk of hospitalisation and long LOS in ED. In addition to its assessment part, ER2 has an intervention part based on tailor-made geriatric recommendations for improved ED care | 1 | 1,698 | 2,233 | Quasi-experimental | System performance Patient outcomes |
| Beauchet et al., 2022, Canada | Neurocognitive disorders | To examine whether the ER2 tool recommendations were associated with incident hospital admissions and LOS in ED in older adults with major neurocognitive disorders visiting ED. | **General assessment and multi-faceted care coordination to reduce avoidable hospital admissions and/or LOS and/or improve ED flow**  ER2 is a validated clinical tool which screens older adults visiting ED at high risk of hospitalisation and long LOS in ED. In addition to its assessment part, ER2 has an intervention part based on tailor-made geriatric recommendations for improved ED care | 1 | 169 | 187 | Quasi-experimental | System performance Patient outcomes |
| Blomaard et al., 2021a, The Netherlands | - | To evaluate the effects of implementation of the APOP screening program for older adults in routine ED care shortly after implementation. | **General assessment and multi-faceted care coordination to reduce avoidable hospital admissions and/or LOS and/or improve ED flow**  The APOP screening program consists of three parts: (1) screening older adults for risk of functional decline/mortality and signs of impaired cognition; (2) targeted interventions for high-risk patients in the ED; and (3) interventions for high-risk patients who are hospitalised or discharged home | 1 | 953 | 920 | Quasi-experimental | System performance Patient outcomes |
| Blomaard et al., 2021b, The Netherlands | - | To explore the experiences with, and attitudes towards geriatric screening in routine ED care among older adults who visited the ED | **General assessment and multi-faceted care coordination to reduce avoidable hospital admissions and/or LOS and/or improve ED flow**  The APOP screening program consists of three parts: (1) screening older adults for risk of functional decline/mortality and signs of impaired cognition; (2) targeted interventions for high-risk patients in the ED; and (3) interventions for high-risk patients who are hospitalised or discharged home | 1 | 13 | - | Qualitative research | Patient experience |
| Bosetti et al., 2020, France | Neurocognitive disorders | To assess the effectiveness of a geriatric emergency medicine unit for elderly patients with neurocognitive disorders admitted to the ED. | **General assessment and multi-faceted care coordination to reduce avoidable hospital admissions and/or LOS and/or improve ED flow**  The Geriatric Emergency Medicine Unit intervention included paramedical assessment by nurses (autonomy, deficiencies, neurosensory disorders, and lifestyle), medical assessment by geriatricians (acute pathology, comorbidities, screening of geriatric syndromes, and regular treatment), and social assessment for patients in a vulnerable social situation. The team developed individual healthcare plans and recommended additional assessments and first treatments. The team guided patients in choosing appropriate care facilities or home care services | 1 | 400 | 401 | Cohort | System performance Patient outcomes |
| Boucher et al., 2019, Canada |  | To compare the acceptability of self-assessment using a tablet in the ED to a standard assessment by a research assistant, according to older adults and their caregivers | **General assessment and multi-faceted care coordination to attenuate functional decline**  Patients were asked to self-assess their functional status using a modified OARS scale, on an electronic tablet. They then assessed their frailty level using a modified CFS | 1 | 67 | - | RCT | Patient experience |
| Brymer et al., 2001, UK |  | To measure the impact of a 1-day workshop for emergency nurses that addresses the educational topics of geriatric depression, physical assessment of the elderly, and mental status testing | **Staff education**  Workshop consisting of case-based didactic sessions addressing (1) physical assessment of the elderly, (2) delirium, depression, and dementia, and (3) a practical approach to mental status testing in the elderly | 1 | 51 | 51 | Pre-post | System performance  Staff experience |
| Callahan et al., 2020, USA | Head trauma | To evaluate the consequences amended trauma activation criteria with a particular interest in the rate of over and under triage | **Geriatric trauma protocol**  Amended trauma activation guidelines requiring at least a Level II activation for all patients 65 years or older taking antithrombotic medication who presented with the suspicion of head trauma | 1 | 398 | 43 | Pre-post | System performance Patient outcomes |
| Carr et al., 2018, USA | Trauma | To identify the age at which an increase in trauma activation status is beneficial | **Geriatric trauma protocol**  A policy change increased the activation criteria to the highest level for patients 70 years or older with a significant mechanism of injury | 1 | 2,422 | 1,919 | Pre-post | System performance Patient outcomes |
| Chong et al., 2021, Singapore | Avoidable admissions | To evaluate the effectiveness of ED Interventions for Frailty in reducing potentially avoidable acute admissions. | **General assessment and multi-faceted care coordination to attenuate functional decline**  Geriatric patients reviewed by Advanced Practice Nurse, and then multidisciplinary geriatric assessment. Follow-up care planning, discharge counselling and education, and telephonic follow-up consultation are provided for those who are discharged. | 1 | 43 | 57 | Quasi-experimental | System performance Patient outcomes |
| Chong et al., 2022, Singapore | Functional  decline post  ED visit | To evaluate the effectiveness of a multicomponent frailty intervention in improving functional outcomes among older persons. | **General assessment and multi-faceted care coordination to attenuate functional decline**  Multicomponent frailty intervention comprising CGA, frailty education, discharge transition package | 1 | 70 | 70 | Quasi-experimental | Patient outcomes |
| Conroy et al., 2014, UK | Avoidable admissions | To evaluate the impact of an embedded CGA service in the ED | **General assessment and multi-faceted care coordination to reduce avoidable hospital admissions and/or LOS and/or improve ED flow**  CGA comprising multidimensional interdisciplinary diagnosis and development of coordinated plan for treatment and follow up | 1 | 9035 | 444 | Pre-post | System performance Patient outcomes |
| Corbett et al., 2005, Australia | Avoidable admissions | To evaluate the effectiveness of the care coordination program operating in the ED in improving outcomes for older adults and reducing ED admissions and re-presentations | **General assessment and multi-faceted care coordination to reduce avoidable hospital admissions and/or LOS and/or improve ED flow**  Care Coordination program | 1 | 134,890 | 40,510 | Pre-post | System performance Patient outcomes |
| Desy et al., 2008, USA | ED nurses  lack of  geriatric-  specific  knowledge | To evaluate the impact of the GENE course on emergency nurses ‘geriatric best practices in the ED | **Staff education**  8-hour Geriatric Emergency Nursing Education course | 1 | 100 | 63 | Pre-post | System performance  Staff experience |
| Dresden et al., 2020, USA | Avoidable admissions | To evaluate the association between GEDI-WISE Transitional Care Nurse care and readmission for geriatric patients who visit the ED within 30 days of a prior hospital discharge | **General assessment and multi-faceted care coordination to reduce avoidable hospital admissions and/or LOS and/or improve ED flow**  ED-based transitional care nurse program to identify individuals with geriatric-specific health-related needs and coordinate their transition from ED to home with the goal of avoiding inpatient admissions when feasible and safe | 3 | 608 | 6230 | Cohort | System performance Patient outcomes |
| Elliott et al., 2017, UK | Utility of  Frailty  screening  tools | To ascertain which validated frailty tools were quick, simple, and acceptable for use in clinical practice. | **General assessment and multi-faceted care coordination to attenuate functional decline**  Frailty screening tools | 1 | 121 | - | Qualitative research | Staff experience |
| Ellis et al., 2012, UK | Avoidable admissions | To evaluate the results of the implementation of an Acute Care for Elders unit situated adjacent to the ED and medical receiving unit | **General assessment and multi-faceted care coordination to reduce avoidable hospital admissions and/or LOS and/or improve ED flow**  Acute Care for Elders unit in the ED | 1 | 210 | 539 | Quasi-experimental | System performance Patient outcomes |
| Fernandez et al., 2019, USA | Trauma | To evaluate outcomes of the T3 implementation | **Geriatric trauma protocol**  A third triage tier introduced for patients with occult head, neck, and torso injury, comprising Emergency Medicine Physician, Trauma Nurse, Laboratory technician, Radiology Technician, and ED Registrar for expedited evaluation of a standard history and physical assessment with emphasis on the primary and secondary trauma survey. The Radiology Department also expedited the acquisition and reading of computed tomography studies ordered under the T3 process | 1 | 3086 | 749 | Pre-post | System performance Patient outcomes |
| Foo et al., 2012 Singapore | ED  Re-attendance | To determine whether geriatric assessment in an ED Observation Unit would have any impact on functional outcomes and health-care utilisation rates | **General assessment and multi-faceted care coordination to reduce avoidable hospital admissions and/or LOS and/or improve ED flow**  Geriatric assessment, immediate interventions initiated such as medication review, or referral to adjunct services such as physio or geriatric outpatients | 1 | 315 | 172 | Quasi-experimental | System performance Patient outcomes |
| Foo et al., 2014, Singapore | Functional decline post ED visit | To determine if risk stratification followed by rapid geriatric screening in an ED reduced functional decline, ED reattendance and hospitalisation. | **General assessment and multi-faceted care coordination to attenuate functional decline**  Screening and assessment, medication reconciliation, postural blood pressure, referral to allied health professionals, geriatric assessment clinic, at home care, and transitional and outreach services as indicated | 1 | 280 | 394 | Quasi-experimental | System performance Patient outcomes |
| Goldberg et al.. 2020a, USA |  | To describe a multidisciplinary team fall prevention intervention for older adults who seek care in the ED after having a fall | **General assessment and multi-faceted care coordination to reduce avoidable hospital admissions and/or LOS and/or improve ED flow**  A brief medication therapy management session delivered by a pharmacist, a fall risk assessment and plan by a physical therapist, referrals to outpatient services (eg, home safety evaluation, outpatient PT). | 2 | 55 | 55 | RCT | System performance Patient outcomes |
| Goldberg et al., 2020b, USA |  | To determine whether an ED–initiated fall-prevention intervention can reduce subsequent fall-related and all cause ED visits and hospital admissions in older adults | **General assessment and multi-faceted care coordination to reduce avoidable hospital admissions and/or LOS and/or improve ED flow**  A brief medication therapy management session delivered by a pharmacist, a fall risk assessment and plan by a physical therapist, referrals to outpatient services (eg, home safety evaluation, outpatient PT). | 2 | 55 | 55 | RCT | Patient outcomes |
| Guttman et al., 2004, Canada | ED Re-attendance | To evaluate the impact of an ED–based nurse discharge plan coordinator on unscheduled return visits within 14 days of discharge, satisfaction with discharge recommendations, adherence with discharge instructions, and perception of well-being of elder patients discharged from the ED | **General assessment and multi-faceted care coordination to reduce avoidable hospital admissions and/or LOS and/or improve ED flow**  Nurse discharge planner provided education, coordinated appointments, telephone follow up | 1 | 819 | 905 | Pre-post | System performance  Patient outcomes  Patient experience |
| Hammer et al., 2016, USA | Trauma | To assess the effect of a change in triage criteria for injured geriatric patients | **Geriatric trauma protocol**  All injured patients over 70 mandated highest level trauma activation comprising attending trauma surgeon, ED physician, surgery and emergency medicine residents, respiratory technologist, three ED nurses, a clinical specialty pharmacist, and representatives from social and chaplain services | 1 | 998 | 1271 | Pre-post | System performance Patient outcomes |
| Heeren et al., 2019 Netherlands | ED Re-attendance | To evaluate the Unplanned Re-admission prevention by Geriatric Emergency Network for Transitional care’ care model | **General assessment and multi-faceted care coordination to reduce avoidable hospital admissions and/or LOS and/or improve ED flow**  Patients at risk identified through screening, those stratified as high risk or identified as needing it, offered CGA to develop a coordinated and integrated plan for treatment and long-term geriatric follow-up, community case management | 1 | 886 | 794 | Quasi-experimental | System performance Patient outcomes |
| Hogan et al., 2016, USA | Pain | To assess the effectiveness of an education and quality improvement program for management of pain in older adults in the ED. | **Assessment and management of pain**  Linked education, EMR tools, and quality improvement techniques, emphasizing how ED staff performance would be measured and expected performance targets. | 1 | 343 | 499 | Pre-post | System performance Patient outcomes |
| Huded et al., 2022, USA |  | To describe the outcomes of GERI-VET, the first comprehensive Veterans Affairs Geriatric ED program | **General assessment and multi-faceted care coordination to better address patient needs**  GERI-VET was designed to identify at-risk older Veterans in the ED, screen for geriatric syndromes, facilitate referrals to aging-friendly resources within and beyond the ED, and assist in care transitions. ED staff performing GERI-VET assessments received education and training as part of the program, equipping them to provide aging-centric care. | 1 | 725 | 725 | Quasi-experimental | System performance Patient outcomes |
| Hullick et al., 2018, Australia | Delirium | This study aimed to determine whether an assistant workforce in the ED could effectively conduct screening to inform assessment and care planning for older adults as well as enhance supportive care activities for prevention of delirium. | **General assessment and multi-faceted care coordination to better address patient needs**  Four full time equivalent OPTAs working 8 h shifts between 8 am to 8 pm in the ED, 7 days a week for 12 months. OPTAs focused on screening and the supportive care of people over the age of 75 years who were not experiencing life threatening situations. | 1 | 3,542 | 8,455 | Pre-post | System performance Patient outcomes Patient experience Staff experience |
| Keene et al., 2022, USA | Avoidable admissions | To determine if assessment and intervention by a GEMA team would decrease the admission rate and reduce the hospital LOS for admitted geriatric patients. | **General assessment and multi-faceted care coordination to reduce avoidable hospital admissions and/or LOS and/or improve ED flow**  The GEMA team consisted of a Geriatric Emergency Medicine-trained Advance Practice Provider (either a nurse practitioner or a physician assistant) and a Geriatric Care Manager, plus a pharmacy technician and occupational therapist, as appropriate. As well as the GEMA team, additional policies and procedures were enacted, including standardisation of discharge paperwork for geriatric patients and additional geriatric continuing medical education requirements for attending physicians and nurses | 1 | 815 | 10,875 | Case-control | System performance Patient outcomes |
| Keyes et al., 2014, USA | Avoidable admissions | To determine whether a new senior ED, caring for patients 65 years and older through case management with specific attention to medication use, activities of daily living, depression, delirium, and alcohol abuse, will result in decreased recidivism, defined as a longer time to return to the ED | **General assessment and multi-faceted care coordination to reduce avoidable hospital admissions and/or LOS and/or improve ED flow**  Opening of a new older person ED in a large community hospital. Changes to the senior ED included staff training, facility redesign, and new processes. Nurses working in the senior ED received a geriatric emergency nursing education course, and physicians completed a web-based curriculum. In the senior ED, physical conditions were modified to include non-skid, non-glare floors, side rails, pressure-reducing mattresses, softer lighting, and larger clocks and televisions. | 1 | 3,748 | 3850 | Pre-post | System performance Patient outcomes |
| Keyes et al., 2019, USA | Head trauma and anticoagulant medication | To determine effectiveness of the “Headstrike” protocol in providing patients with timely treatment and disposition, while maintaining positive outcomes. | **Management of anticoagulated older adult with head injury**  Protocol for anticoagulated older adults suffering ground-level falls. The response team for the “Headstrike” tier would consist of all the staff for full level II trauma activations, except for the trauma surgical team. This would allow expeditious, high-quality care, while simultaneously using trauma service line resources more efficiently. If the patient met the higher trauma activation criteria based on other physiologic or anatomic criteria, there would be an immediate activation upgrade, with the trauma surgical team required to be present within 15 minutes. | 1 | 515 | - | Cohort | System performance Patient outcomes |
| Lee et al., 2001, Canada |  | To describe a rehabilitation consultation service and present results of an evaluative study of 80 patients who received comprehensive functional assessments in the ED | **General assessment and multi-faceted care coordination to better address patient needs**  Occupational and physical therapist-led rehabilitation consultation service for the ED. A functional safety checklist guided emergency staff in identifying patients in need of comprehensive functional assessment based on transfers, ambulation, activities of daily living tasks and cognition. A comprehensive assessment was conducted by Occupational Therapist or Physical Therapist. Decisions regarding appropriate discharge plans and destinations were made in consultation with emergency staff, and arrangements made for necessary community resources for patients discharged home. | 1 | 80 | - | Cohort | Patient outcomes |
| Lesser et al., 2018, USA | Functional decline post ED visit | To determine whether providing PT services in the ED improves outcomes for older adults who fall | **General assessment and multi-faceted care coordination to attenuate functional decline**  PT services in ED | Unclear | 17,975 | 179,950 | Cohort | System performance Patient outcomes |
| Liberman et al., 2018, USA | Unmet need/palliative care | To investigate the effectiveness of our Advanced Illness Management program in the ED on the 1). % of patients identified with advanced illness; 2) % of patients who had GOC conversations; and 3) % of patients who were referred to home hospice | **Palliative or supportive care**  Screen ED patients for advanced illness, provide ED health care providers with training to have goals-of-care discussions, conduct ED-led goals-of-care promptly, and develop a safe discharge to hospice for appropriate patients. An interdisciplinary team consisting of nurses, emergency and palliative care physicians, social workers, case management, quality, and administration was formed and met bi-weekly during the study period | 1 | 61 | 21 | Pre-post | System performance  Patient outcomes Patient experience |
| Liberman et al., 2020, USA | To improve care of older adults in the ED | To determine whether identifying older adults that could be safely sent home with connection to community resources decreased 30-day revisit and hospitalisation rates and achieved high patient and family satisfaction | **Palliative or supportive care**  Implementation of a Geriatrics and Palliative Medicine ED team comprised of a Geriatric and Palliative Medicine physician, an Emergency physician, a Social Worker specialised in Geriatrics and Palliative Medicine, nursing leadership, and administrative and research support. | 1 | 283 | 283 | Pre-post | Patient outcomes  Patient experience |
| Liu et al., 2019, Taiwan | PIMs/polypharmacy | To determine effect of medication reconciliation on polypharmacy for older adults in ED | **Medication management**  Computer-based and pharmacist-assisted medication review initiated in the ED | 1 | 668 | 243 | Pre-post | System performance  Patient outcomes |
| Liu et al., 2021, Sweden | Avoidable admissions | To investigate whether a dedicated interprofessional emergency team also can reduce the hospital admission rate without the resources required by the formal use of CGA | **General assessment and multi-faceted care coordination to reduce avoidable hospital admissions and/or LOS and/or improve ED flow**  One interprofessional team non-geriatric specialists and an older-friendly area to improve the care for older patient | 1 | 634 | 3,950 | Pre-post | System performance  Patient outcomes |
| Mahony et al., 2008, USA | Unmet need/palliative care | To evaluate the impact of a project to identify chronically ill older adults presenting to ED in need of palliative care, homecare, and hospice services and to increase linkage with these services | **Palliative or supportive care**  Two palliative care nurse practitioners in ED Mon-Fri 1100-2100, identify patients for pal care, conduct clinical consultation and linked to homecare and hospice services. Also educates ED staff on indications for pal care referral and service access. | 1 | 291 | 125 | Descriptive | System performance Patient experience |
| Marsden et al., 2022, Australia | Avoidable admissions | To evaluate the healthcare outcomes and costs associated with the implementation of the GEDI for adults aged 70 years and over at two hospital sites | **General assessment and multi-faceted care coordination to better address patient needs**  Geriatric ED - nurse-led intervention to improve health outcomes for frail older adults in ED using InterRAI ED screener | 2 | 19,248 | 18,324 | Pre-post | System performance Patient outcomes |
| Matz et al., 2021, Germany |  | To compare telemedical assessment of geriatric patients in the ED with ED standard treatment with particular focus on PIMs | **Medication management**  An instant telemedical consultation was carried out by a geriatrician independently of the standard assessment by the ED medical staff | 1 | 50 | - | Cohort | System performance Patient outcomes |
| McCusker et al., 2001, Canada | Functional decline post ED visit | To determine the effectiveness of a two-stage (screening and nursing assessment) intervention for older adults in the ED who are at increased risk of functional decline and other adverse outcomes | **General assessment and multi-faceted care coordination to attenuate functional decline**  Brief, standardised geriatric nursing assessment, and referrals to primary physician, community health centre, geriatric outpatient clinic, and other community services | 4 | 178 | 210 | RCT | System performance Patient outcomes |
| McCusker et al., 2003a, Canada | Functional decline post ED visit | To determine the cost of a two-stage (screening and nursing assessment) intervention for older adults in the ED who are at increased risk of functional decline and other adverse outcomes | **General assessment and multi-faceted care coordination to attenuate functional decline**  Brief, standardised geriatric nursing assessment, and referrals to primary physician, community health centre, geriatric outpatient clinic, and other community services | 4 | 178 | 210 | RCT | System performance Patient outcomes |
| McCusker et al., 2003b, Canada | Functional decline post ED visit | To investigate the process of care during the ED visit and the following month, to understand better those care processes that were responsible for the cost–effectiveness of the nursing assessment and intervention in ED | **General assessment and multi-faceted care coordination to attenuate functional decline**  Brief, standardised geriatric nursing assessment, and referrals to primary physician, community health centre, geriatric outpatient clinic, and other community services | 4 | 178 | 210 | RCT | System performance |
| McGrath et al., 2019, UK |  | To describe the CGA ED intervention and assess whether it met its aims to increase % of older adults assessed using CFS and decrease admissions | **General assessment and multi-faceted care coordination to better address patient needs**  CGA comprising CFS, ambulatory care frailty pathway, championing the CGA | 1 | 319 | - | Time series | System performance  Patient experience  Staff experience |
| Miller et al., 1996, USA | Functional decline post ED visit | To evaluate the effects of a program of case-finding and liaison service for older adults | **General assessment and multi-faceted care coordination to attenuate functional decline**  Geriatric assessment, immediate interventions, including further work up, recommendations to family for e.g. follow-up with family physician, specialist, social and dental services, and appointments made | 1 | 385 | 385 | Case-control | System performance Patient outcomes |
| Moss et al., 2019, USA | PIMs/polypharmacy | To evaluate the impact of an academic detailing intervention on (1) self-reported confidence in prescribing for older adults and (2) rates of PIMs prescribed to older adults by physician residents in a Veteran Affairs ED | **Medication management**  An academic detailing intervention delivered as part of a quality improvement project by a physician–pharmacist pair | 1 | 63 | 67 | Quasi-experimental | System performance Staff experience |
| Newton-Brown et al., 2014, Australia | Pain | To test the hypothesis that the implementation of educational and awareness strategies increases knowledge and implementation of evidence-based use of nerve blocks as a mode of analgesia for elderly patients with a fractured neck of femur in ED. | **Assessment and management of pain**  Education aligned with practice deficiencies identified by chart audit, delivered over 2-week period, specific care plan, 90-min teaching session on nerve blocks - use of ultrasound guidance, Ropivacaine, facia iliac approach, and implementation of a pack containing consumables and documentation requirements, prompts to consider nerve block for analgesia | 1 | 66 | 70 | Pre-post | System performance Patient outcomes |
| Ngian et al., 2008, Australia |  | To review discordant cases of elderly patients deemed for discharge by ED but subsequently admitted following Aged Care Service Emergency Teams review | **General assessment and multi-faceted care coordination to better address patient needs**  An on-call senior geriatrician supervising a Geriatric Medicine trainee based solely in ED. The operating hours are 10 am to 6 pm weekdays and 10 am to 4 pm weekends. The service is supported by on-site nursing and allied health team comprising of a physiotherapist, occupational therapist, and social worker. Post-discharge follow-up facilities include falls, memory, and general outpatient clinics. | 1 | 103 | - | Cross-sectional | System performance Patient outcomes |
| O' Keeffe et al., 2020, Ireland | Falls | To evaluate a Nurse Practitioner led ED falls pathway for older adults | **Management of falls risk**  Investigate modifiable falls risk assessment comprising postural blood pressure, 4AT, TUG, polypharmacy (>5), stratify, medium/high risk also get ECG, urinalysis, and clinically indicated bloods; high risk reviewed by senior doctor or nurse, for discharged patients -streamlined referral for appropriate community follow-up | 1 | 77 | 59 | Pre-post | System performance Patient outcomes |
| O'Grady et al., 1996 , Australia |  | To Evaluate the Quick Response Program for older adults in ED | **General assessment and multi-faceted care coordination to better address patient needs**  Community health nurse with gerontological training based in ED from 8:30 am to 7 pm assessed identified patients, liaised with ED and GPs and developed care plans, referred to community services, advocated for best patient outcome | 1 | NR | NR | Descriptive | System performance Patient outcomes Patient experience Staff experience |
| Palonen et al., 2015, Finland | Discharge education | To examine the association of discharge education with discharge readiness among older adults and their family members in an emergency setting in Finland | **General assessment and multi-faceted care coordination to better address patient needs**  Discharge education | 2 | 135 patients; 128 family members | - | Cross-sectional | System performance Patient outcomes |
| Pelaez et al., 2021, USA | Head Trauma Patients on Anticoagulants | To evaluate a hospital initiative to improve the trauma triage response for injured elderly patients taking anticoagulation or antiplatelet medications with suspected head injury. | **Management of anticoagulated older adult with head injury**  Revised trauma response criteria: patients taking anticoagulation or antiplatelet medications with evidence of head injury received a rapid ED response | 1 | 142 | 91 | Pre-post | System performance Patient outcomes |
| Puig Campmany et al., 2019, Spain | Frailty | To present how a Program of Care for Frailty in an ED impact on patient flows | **General assessment and multi-faceted care coordination to attenuate functional decline**  Program of Care for Frailty: (1) inclusion of CGA procedures by ED staff; (2) a joint project to strengthen the connections circuits with post-hospital care providers, allowing direct and early referral from the ED to post-acute care hospitals, home hospitalisation, nursing homes and others; (3) a new area that is structurally and functionally adapted to the frailer patients | 1 | 295,260 | - | Descriptive | System performance Patient outcomes |
| Rittenhouse et al., 2015, USA | Head Trauma Patients on Anticoagulants | To determine if a novel ED protocol prioritises workup and improves outcome | **Management of anticoagulated older adult with head injury**  ACT Alert Response protocol - initiates three procedures in the ED: workup by an ED response team within 15 minutes, international normalised ratio test within 20 minutes, and STAT priority head CT scan within 30 minutes | 1 | 415 | 337 | Quasi-experimental | System performance Patient outcomes |
| Scarpazza et al., 2008, Italy | Acute respiratory failure | To assess the outcome of non-invasive mechanical ventilation in a group of elderly patients with acute hypercapnic acute respiratory failure who had a do-not-intubate status | **Non-invasive mechanical ventilation** | 1 | 62 | - | Cohort | System performance  Patient outcomes |
| Shaw et al., 2003, UK | Cognitive impairment and dementia | To determine the effectiveness of multifactorial intervention after a fall in older adults with cognitive impairment and dementia attending the accident and emergency department | **Management of falls risk**  A multifactorial assessment and intervention protocol, which comprised assessment, risk factor identification, and interventions, n four areas of care (medical, cardiovascular, physiotherapy, and occupational therapy) | 2 | 150 | 158 | RCT | System performance Patient outcomes |
| Silvester et al., 2014, UK |  | To identify and address delays in ensuring timely care, without additional resources | **General assessment and multi-faceted care coordination to reduce avoidable hospital admissions and/or LOS and/or improve ED flow**  Diagnostic patient flow analysis followed by a series of Plan Do Study Act cycles to test and implement changes by a multidisciplinary team using time series run charts | 1 | 16,953 | - | Time series | System performance Patient outcomes |
| Southerland et al., 2018, UK |  | To investigate the effects of offering multidisciplinary assessments for ED patients in an ED observation unit | **General assessment and multi-faceted care coordination to better address patient needs**  Evaluation by a geriatric hospital consultation team, physical therapist, case manager, and/or pharmacist | 1 | 89 | 132 | Quasi-experimental | System performance Patient outcomes |
| Stevens et al., 2017, USA | PIMs/polypharmacy | To evaluate the effectiveness and sustainability of EQUiPPED to reduce the use of PIMs prescribed to older Veterans at the time of ED discharge | **Medication management**  EQUiPPED – combining education, electronic health record based clinical decision support tools, and individual provider audit and feedback with peer benchmarking | 4 | NR | NR | Pre-post | System performance Patient outcomes |
| Tousignant-Laflamme et al., 2015, Canada | IS | To assess the feasibility and explore the potential clinical value of adding PT services to the ED, in collaboration with nursing staff, to prevent immobilisation syndrome | **General assessment and multi-faceted care coordination to attenuate functional decline**  PT services in the ED | 1 | 9 | 11 | Descriptive | Patient outcomes |
| Travers et al., 2021 , USA | Head trauma and anticoagulant medication | To assess the impact of the novel level III trauma protocol on resource utilisation and patient outcome | **Management of anticoagulated older adult with head injury**  The level III trauma protocol specified that all patients aged ≥ 65 years old who were taking any anticoagulants or antiplatelet agents and presenting to the ED after a fall with head injury would be immediately evaluated by a physician | 1 | 12 | 56 | Quasi-experimental | System performance Patient outcomes |
| van der Zwaard et al., 2020, The Netherlands | Hip fracture | To compare the number of older adults with hip fractures who opted for non-surgical management after the addition of pre-operative CGA with shared decision making by a geriatrician to usual care | **Assessment and management of hip fracture**  Pre-operative CGA: geriatrician saw the patient pre-operatively and determined psychological, social, and functional status of the patient in addition to the medical status. Subsequently, a treatment plan and plan for follow-up were tailored for patients during pre-operative CGA using shared decision making involving the geriatrician, the patient, and/or their representatives, or family. | 1 | 224 | 206 | Quasi-experimental | Patient outcomes |
| Vaughan et al., 2021, USA | PIMs/polypharmacy | To describe adaptation of EQUIPPED from VA to non-VA academic health centres and early prescribing outcomes | **Medication management**  EQUIPPED: combining education, electronic health record based clinical decision support tools, and individual provider audit and feedback with peer benchmarking | 3 | NR | NR | Pre-post | System performance Patient outcomes |
| Vivanti et al., 2015, Australia | Malnutrition | To trial malnutrition screening in older adults presenting to an ED and compare two service delivery models of nutritional support on nutritional status, quality of life, falls and unplanned hospital admissions | **General assessment and multi-faceted care coordination to better address patient needs**  Individualised dietary counselling in which nutrition goals and strategies were made in collaboration with the ED dietitian | 1 | 10 | 14 | RCT | System performance Patient outcomes |
| Wallace et al., 2019, USA | Hip fracture | To evaluate the effects of a multidisciplinary hip fracture care pathway on patient outcomes in the care of elderly patients | **Assessment and management of hip fracture**  An evidence-based clinical practice guideline for the management of hip fractures to optimize patient care and surgical outcomes | 1 | 191 | 80 | Pre-post | Patient outcomes System performance |
| Wallis et al., 2018, Australia | Avoidable admissions | To evaluate a GEDI model of service delivery for adults aged 70 years and older. | **General assessment and multi-faceted care coordination to better address patient needs**  A nurse-led, physician-championed, ED intervention to improve the care of frail older adults in the ED. The nurses had gerontology experience and education and provided targeted geriatric assessment and streamlining of care | 1 | 25,675 | 19,308 | Pre-post | System performance Patient outcomes |
| Warburton et al., 2005, Canada | Improve outcomes for patients aged 75 or more visiting the Emergency Department (ED) | To assess outcomes of identifying older adults at risk of adverse outcomes of hospitalization, plan interventional care, and provide targeted preventative services. | **General assessment and multi-faceted care coordination to better address patient needs**  Screen for risk using ISAR tool, interventional care planning | 1 | 127, stratified into screened, no referrals (HP)n= 34; screened, received all services (HC) n = 38; screened, low risk (L) n = 55 | 150 | Quasi-experimental | System performance Patient outcomes |
| Wiles et al., 2018, USA | Trauma | To determine whether the institution of a third-tier trauma protocol results in a change in geriatric trauma patient outcomes, complications, and mortality rates | **Geriatric trauma protocol**  Delta alert to expand criteria for geriatric trauma patients | 1 | 125 | 62 | Pre-post | System performance Patient outcomes |
| Wright et al., 2014, UK |  | To measure the effect of TREAT on LOS and the rate of same-day discharges | **Geriatric trauma protocol**  TREAT – combining early Accident and Emergency-based senior doctor review, CGA, therapist assessment and supported discharge; post-discharge supported recovery; and a rapid access geriatric ‘hot-clinic’. TREAT was supported by a post-acute care enablement team, providing short-term nursing support immediately following discharge. | 1 | 3,322 | 3,084 | Pre-post | System performance Patient outcomes |

APOP: acutely presenting older patient. APRN: Advanced Practice Registered Nurses. CFS: Clinical Frailty Scale. COPE: comprehensive older person’s evaluation. CGA: comprehensive geriatric assessment. ED: Emergency Department. EMR: electronic medical record. EQUiPPED: Enhancing Quality of Provider Practices for Older Adults in the Emergency Department. ER2: Emergency Room Evaluation and Recommendations. GEDI: Geriatric Emergency Department Intervention. GEDI WISE: Geriatric Emergency Department Innovations in Workforce, Informatics, and Structural Enhancements. GEMA: Geriatric Emergency Medicine Assessment. GERI-VET: Geriatric Emergency Room Innovations for Veterans. ISAR: Identification of Seniors at Risk. LOS: length of stay. PIM: potentially inappropriate medication. PT: physical therapy. T3: Expedited Emergency Medicine Physician evaluation protocol. TREAT: Triage and Rapid Elderly Assessment Team.
