# Supplementary material for: Strategies to improve care for older adults who present to the emergency department: a systematic review
Source: BMC Health Serv Res. 2024 Feb 8;24:178. doi: 10.1186/s12913-024-10576-1 (PMC10851482; doi:10.1186/s12913-024-10576-1)
Supplement: Supplementary file 3 — Additional file 3: Supplement 3. JBI Critical Appraisal Checklists for included studies. [file 12913_2024_10576_MOESM3_ESM.docx]

**Supplement 3. JBI Critical Appraisal Checklists for included studies**

1. **Randomised controlled trials**

| Authors | Year | Was true randomization used for assignment of participants to treatment groups? | Was allocation to treatment groups concealed? | Were treatment groups similar at the baseline? | Were participants blind to treatment assignment? | Were those delivering treatment blind to treatment assignment? | Were outcomes assessors blind to treatment assignment? | Were treatment groups treated identically other than the intervention of interest? | Was follow up complete and if not, were differences between groups in terms of their follow up adequately described and analyzed? | Were participants analyzed in the groups to which they were randomized? | Were outcomes measured in the same way for treatment groups? | Were outcomes measured in a reliable way? | Was appropriate statistical analysis used? | Was the trial design appropriate, and any deviations from the standard RCT design accounted for in the conduct and analysis of the trial? | Overall appraisal |
| --- | --- | --- | --- | --- | --- | --- | --- | --- | --- | --- | --- | --- | --- | --- | --- |
| Basic et al. | 2005 | Yes | Yes | No | No | No | No | Yes | Yes | Unclear | Yes | Yes | Yes | Yes | Include |
| Boucher et al. | 2019 | Yes | No | N/A | N/A | No | No | Yes | Yes | N/A | Yes | Yes | Yes | Yes | Include |
| Goldberg et al. | 2020a | Yes | Yes | Yes | Unclear | No | Unclear | Yes | Yes | No | Yes | Yes | Unclear | Unclear | Include |
| Goldberg et al. | 2020b | Yes | Yes | Yes | Unclear | Unclear | Yes | Yes | Yes | No | Yes | Yes | Yes | Yes | Include |
| McCusker et al. | 2001 | Yes | No | Yes | No | No | No | Yes | Yes | Yes | Yes | Yes | Yes | Yes | Include |
| McCusker et al. | 2003a | Yes | No | No | No | No | No | Yes | No | Yes | Yes | Yes | Yes | Yes | Include |
| McCusker et al. | 2003b | Yes | No | Unclear | No | No | No | Yes | Yes | Yes | Yes | Yes | Yes | Yes | Include |
| Shaw et al. | 2003 | Yes | Yes | Yes | No | No | Yes | Yes | Yes | Yes | Yes | Yes | Yes | Yes | Include |
| Vivanti et al. | 2015 | Yes | Yes | Unclear | No | No | No | Yes | Unclear | Yes | Yes | Yes | Yes | No | Include |

1. **Quasi-Experimental and non-randomised studies**

| Authors | Year | Is it clear in the study what is the ‘cause’ and what is the ‘effect’ (i.e. there is no confusion about which variable comes first)? | Were the participants included in any comparisons similar? | Were the participants included in any comparisons receiving similar treatment/care, other than the exposure or intervention of interest? | Was there a control group? | Were there multiple measurements of the outcome both pre and post the intervention/exposure? | Was follow up complete and if not, were differences between groups in terms of their follow up adequately described and analyzed? | Were the outcomes of participants included in any comparisons measured in the same way? | Were outcomes measured in a reliable way? | Was appropriate statistical analysis used? | Overall appraisal |
| --- | --- | --- | --- | --- | --- | --- | --- | --- | --- | --- | --- |
| Ageron et al. | 2016 | Yes | Unclear | Unclear | Yes | Yes | Yes | Yes | Yes | Yes | Include |
| Aldeen et al. | 2014 | Yes | No | Yes | Yes | Yes | Yes | Yes | Yes | Yes | Include |
| Arendts et al. | 2013 | Yes | No | Yes | Yes | Yes | Yes | Yes | Yes | Yes | Include |
| Arendts et al. | 2020 | Yes | Yes | Yes | Yes | No | Yes | Yes | Yes | Yes | Include |
| Beauchet et al. | 2021 | Yes | Yes | Yes | Yes | Yes | Yes | Yes | Yes | Yes | Include |
| Beauchet et al. | 2022 | Yes | Yes | Yes | Yes | Yes | Yes | Yes | Yes | Yes | Include |
| Blomaard et al. | 2021a | Yes | Yes | No | Yes | Yes | Yes | Yes | Yes | Yes | Include |
| Brymer et al. | 2001 | Yes | Yes | Yes | Yes | Yes | Yes | Yes | No | Yes | Include |
| Callahan et al. | 2020 | Yes | No | Yes | Yes | No | Yes | Yes | Yes | Yes | Include |
| Carr et al. | 2018 | Yes | No | Yes | Yes | Yes | Yes | Yes | Yes | Yes | Include |
| Chong et al. | 2021 | Yes | No | Yes | Yes | Yes | Yes | Yes | Yes | Yes | Include |
| Chong et al. | 2022 | Yes | Yes | Yes | Yes | Yes | Yes | Yes | Yes | Yes | Include |
| Conroy et al. | 2014 | Yes | Unclear | Yes | Yes | Yes | Yes | Yes | Yes | Yes | Include |
| Corbett et al. | 2005 | Yes | Yes | Unclear | Yes | Yes | Unclear | Yes | Yes | Yes | Include |
| Desy et al. | 2008 | Yes | Yes | Yes | Yes | Yes | Yes | Yes | Yes | Yes | Include |
| Ellis et al. | 2012 | Yes | Yes | No | Yes | Yes | Yes | Yes | Yes | Yes | Include |
| Fernandez et al. | 2019 | Yes | No | Yes | Yes | Yes | Yes | Yes | Yes | Yes | Include |
| Foo et al. | 2012 | Yes | Yes | Yes | Yes | Yes | Yes | Yes | Yes | Yes | Include |
| Foo et al. | 2014 | Yes | No | Yes | Yes | Yes | Yes | Yes | Yes | Yes | Include |
| Guttman et al. | 2004 | Yes | No | Yes | Yes | Yes | Unclear | Yes | No | yes | Include |
| Hammer et al. | 2016 | Yes | No | Yes | Yes | Yes | N/A | Yes | Yes | Yes | Include |
| Heeren et al. | 2019 | Yes | No | Yes | Yes | Yes | Yes | Yes | Yes | Yes | Include |
| Hogan et al. | 2016 | Yes | Yes | Yes | Yes | Yes | Yes | Yes | Yes | Yes | Include |
| Huded et al. | 2022 | Yes | No | Yes | Yes | Yes | Yes | Yes | Yes | Yes | Include |
| Hullick et al. | 2018 | Yes | Yes | Yes | Yes | Yes | Yes | Yes | Yes | Yes | Include |
| Keyes et al. | 2014 | Yes | Yes | Yes | Yes | Yes | Yes | Yes | Yes | Yes | Include |
| Liberman et al. | 2018 | Yes | No | Yes | Yes | Yes | Yes | Yes | Yes | Yes | Include |
| Liberman et al. | 2020 | Yes | Yes | Yes | Yes | No | Yes | Yes | No | Yes | Include |
| Liu et al. | 2019 | Yes | No | Unclear | Yes | Yes | Yes | Yes | No | Yes | Include |
| Liu et al. | 2021 | Yes | No | Unclear | Yes | Yes | Yes | Yes | Yes | Yes | Include |
| Marsden et al. | 2022 | Yes | Unclear | Yes | Yes | No | Yes | Yes | Yes | Yes | Include |
| McGrath et al. | 2019 | Yes | Unclear | Yes | Yes | Yes | Yes | Yes | Yes | Yes | Include |
| Moss et al. | 2019 | Yes | Yes | Yes | Yes | Yes | No | Yes | Yes | Yes | Include |
| Newton-Brown et al. | 2014 | Yes | Unclear | Unclear | Yes | Yes | Yes | Yes | Yes | No | Include |
| O' Keeffe et al. | 2020 | Yes | Unclear | Unclear | Yes | No | No | No | Yes | No | Include |
| Pelaez et al. | 2021 | Yes | Yes | yes | Yes | Yes | Yes | Yes | Yes | Yes | Include |
| Rittenhouse et al. | 2015 | Yes | No | Yes | Yes | Yes | Yes | Yes | Yes | No | Include |
| Silvester et al. | 2014 | Yes | Unclear | Yes | Yes | yes | No | Yes | Yes | Yes | Include |
| Southerland et al. | 2018 | Yes | Unclear | Yes | Yes | Yes | No | Yes | Yes | Yes | Include |
| Stevens et al. | 2017 | Yes | Unclear | Yes | Yes | No | Unclear | Yes | Yes | Yes | Include |
| Travers et al. | 2021 | Yes | Yes | Yes | Yes | Yes | Yes | Yes | Yes | Yes | Include |
| van der Zwaard et al. | 2020 | Yes | Yes | Yes | Yes | Yes | Yes | Yes | Yes | Yes | Include |
| Vaughan et al. | 2021 | Yes | Yes | Yes | Yes | Yes | Yes | Yes | Yes | Yes | Include |
| Wallace et al. | 2019 | Yes | No | Yes | Yes | Yes | N/A | Yes | Yes | Yes | Include |
| Wallis et al. | 2018 | Yes | Yes | Yes | Yes | Yes | Yes | Yes | Yes | Yes | Include |
| Warburton et al. | 2005 | Yes | Yes | Yes | Yes | Yes | Yes | Yes | Unclear | Yes | Include |
| Wiles et al. | 2018 | Yes | Yes | Yes | Yes | Yes | Yes | Yes | Yes | Yes | Include |
| Wright et al. | 2014 | Yes | Yes | Yes | Yes | Yes | Yes | Yes | Yes | Yes | Include |

1. **Cohort studies**

| **Authors** | **Year** | **Were the two groups similar and recruited from the same population?** | **Were the exposures measured similarly to assign people to both exposed and unexposed groups?** | **Was the exposure measured in a valid and reliable way?** | **Were confounding factors identified?** | **Were strategies to deal with confounding factors stated?** | **Were the groups/participants free of the outcome at the start of the study (or at the moment of exposure)?** | **Were the outcomes measured in a valid and reliable way?** | **Was the follow up time reported and sufficient to be long enough for outcomes to occur?** | **Was follow up complete, and if not, were the reasons to loss to follow up described and explored?** | **Were strategies to address incomplete follow up utilized?** | **Was appropriate statistical analysis used?** | **Overall appraisal** |
| --- | --- | --- | --- | --- | --- | --- | --- | --- | --- | --- | --- | --- | --- |
| Argento et al. | 2014 | Yes | Yes | Yes | No | No | Yes | Unclear | Yes | Unclear | Unclear | Yes | Include |
| Bosetti et al. | 2020 | No | Yes | Yes | Yes | Yes | Yes | Yes | Yes | Yes | N/A | Yes | Include |
| Dresden et al. | 2020 | Unclear | Unclear | Unclear | Unclear | No | Yes | Yes | Yes | Yes | N/A | No | Include |
| Keyes et al. | 2019 | N/A | N/A | Yes | No | No | Yes | Yes | Yes | Yes | No | Yes | Include |
| Lee et al. | 2001 | N/A | N/A | Yes | No | No | Yes | Yes | No | No | No | No | Include |
| Lesser et al. | 2018 | No | Yes | Yes | Yes | Yes | Yes | Yes | Yes | Yes | Yes | Yes | Include |
| Matz et al. | 2021 | Yes | Yes | Yes | Unclear | No | Yes | Yes | Yes | Yes | N/A | Yes | Include |
| Scarpazza et al. | 2008 | Yes | Unclear | Unclear | No | N/A | Yes | Yes | Yes | Yes | N/A | Unclear | Include |

1. **Cross-sectional studies**

| **Authors** | **Year** | **Were the criteria for inclusion in the sample clearly defined?** | **Were the study subjects and the setting described in detail?** | **Was the exposure measured in a valid and reliable way?** | **Were objective, standard criteria used for measurement of the condition?** | **Were confounding factors identified?** | **Were strategies to deal with confounding factors stated?** | **Were the outcomes measured in a valid and reliable way?** | **Was appropriate statistical analysis used?** | **Overall appraisal** |
| --- | --- | --- | --- | --- | --- | --- | --- | --- | --- | --- |
| Ngian et al. | 2008 | Yes | Yes | Yes | Yes | Yes | No | Yes | Yes | Include |
| Palonen et al. | 2015 | Yes | Yes | Yes | No | No | No | No | Yes | Include |

1. **Case-control studies**

| **Authors** | **Year** | **Were the groups comparable other than the presence of disease in cases or the absence of disease in controls?** | **Were cases and controls matched appropriately?** | **Were the same criteria used for identification of cases and controls?** | **Was exposure measured in a standard, valid and reliable way?** | **Was exposure measured in the same way for cases and controls?** | **Were confounding factors identified?** | **Were strategies to deal with confounding factors stated?** | **Were outcomes assessed in a standard, valid and reliable way for cases and controls?** | **Was the exposure period of interest long enough to be meaningful?** | **Was appropriate statistical analysis used?** | **Overall appraisal** |
| --- | --- | --- | --- | --- | --- | --- | --- | --- | --- | --- | --- | --- |
| Keene et al. | 2022 | No | Yes | Yes | Yes | Yes | No | Yes | Yes | Yes | Yes | Include |
| Miller et al. | 1996 | Yes | Yes | No | Yes | Yes | No | No | No | Yes | No | Include |

1. **Qualitative studies**

| **Authors** | **Year** | **Is there congruity between the stated philosophical perspective and the research methodology?** | **Is there congruity between the research methodology and the research question or objectives?** | **Is there congruity between the research methodology and the methods used to collect data?** | **Is there congruity between the research methodology and the representation and analysis of data?** | **Is there congruity between the research methodology and the interpretation of results?** | **Is there a statement locating the researcher culturally or theoretically?** | **Is the influence of the researcher on the research, and vice- versa, addressed?** | **Are participants, and their voices, adequately represented?** | **Is the research ethical according to current criteria or, for recent studies, and is there evidence of ethical approval by an appropriate body?** | **Do the conclusions drawn in the research report flow from the analysis, or interpretation, of the data?** | **Overall appraisal** |
| --- | --- | --- | --- | --- | --- | --- | --- | --- | --- | --- | --- | --- |
| Blomaard et al. | 2021b | Yes | Yes | Yes | Yes | Yes | No | Yes | Yes | Yes | Yes | Include |
| Elliott et al. | 2017 | No | No | Yes | Yes | Yes | No | No | Yes | Yes | Yes | Include |
